# Rev‐erb‐α in Skeletal Muscle Adaptation: Integrating Circadian Control Mechanisms and Exercise Dynamic Responses

**DOI:** 10.1002/jcp.70239

**Published:** 2026-10-09

**Authors:** Ivo Vieira de Sousa Neto, Alisson Luiz da Rocha, Ana Paula Pinto, Eliézer Lucas Pires Ramos, Eduardo Rochete Ropelle, José Rodrigo Pauli, Ellen Cristini de Freitas, Adelino Sanchez Ramos da Silva

**Affiliations:** ^1^ School of Physical Education and Sport of Ribeirão Preto University of São Paulo (USP) Ribeirão Preto São Paulo Brazil; ^2^ School of Applied Sciences University of Campinas (UNICAMP) Limeira São Paulo Brazil; ^3^ Department of Health Sciences, Ribeirão Preto Medical School University of São Paulo (USP) Ribeirão Preto São Paulo Brazil; ^4^ Postgraduate Program in Rehabilitation and Functional Performance, Ribeirão Preto Medical School University of São Paulo (USP) Ribeirão Preto São Paulo Brazil

**Keywords:** autophagy, circadian rhythms, exercise, metabolic flexibility, myogenesis

## Abstract

Circadian rhythms orchestrate a host of biological processes in a time‐dependent manner to maintain homeostasis in response to exercise. Within this framework, Rev‐erb‐α, an intracellular nuclear receptor and transcriptional regulator, transcends its role in circadian rhythms to drive myogenesis, muscle remodeling, primary metabolism, and autophagy, underscoring its versatile, pleiotropic actions. This review consolidates experimental evidence from cell culture, genetically modified animal models, drug‐based approaches and humans, highlighting the role of Rev‐erb‐α mechanisms in skeletal muscle and exercise adaptation. Recent frontier‐of‐knowledge data indicate that acute and chronic exercise elicit distinct Rev‐erb‐α signatures. There is bimodal regulation: Rev‐erb‐α is downregulated immediately post‐exercise, followed by a subsequent increase, reflecting its primary role in pathway repression. In chronic conditions, the upregulation of Rev‐erb‐α can act as an adaptive mechanism that governs robust responses to energy demand and mitochondrial function, thereby coordinating the metabolic network, calcium homeostasis, and oxidative capacity. Also, Rev‐erb‐α overexpression or its pharmacological activation may reduce pathways associated with muscle atrophy, thereby preserving tissue function and muscle mass. Excitingly, higher physical activity levels increase the amplitude of Rev‐erb‐α over 24 h, enhancing health and performance. However, exercise timing determines the Rev‐erb‐α signature that affects downstream phenotypes in skeletal muscle. Thus, elucidating the mechanisms of regulation and action of the Rev‐erb‐α protein may offer new perspectives for improving or preserving muscle quality across different health conditions and diseases.

## 1 Introduction

Circadian rhythms are intrinsic 24‐h cycles that temporally organize behavior and physiological processes, thereby promoting homeostasis and adaptive responses (Sieck 2021). Governed by cellular clocks dispersed throughout the organism, these rhythms regulate the mammalian adaptation by priming the brain and other peripheral tissues, including skeletal muscle (Hodge et al. 2015). Receptors in the retina are sensitive to light:dark cycles and communicate information to the suprachiasmatic nucleus (SCN), which modulates phase settings and consequently variations in many biological functions, such as sleep, blood pressure, body temperature, hormone levels, and whole‐body metabolism (Patke et al. 2020). In addition to their role in sustaining clock oscillation, these elements also regulate the extensive gene activation beyond the circadian feedback loop (Fagiani et al. 2022). The resultant targets, known as clock‐controlled genes, exhibit significant tissue specificity and dictate particular physiological outcomes, including transcription, cell signaling, and energy metabolism (Everett and Lazar 2014).

The clock genomic machinery has been characterized in skeletal muscles, revealing that over 1000 genes exhibit rhythmic expression (Hodge et al. 2015; R. Zhang et al. 2014). Mechanistically, the primary system is composed of transcriptional activators, such as Circadian Locomotor Output Cycles Kaput (Clock) and basic helix‐loop‐helix Arnt‐like 1 (Bmal1), which act by binding to E‐box elements in the promoter regions of target genes, including period circadian clock 1 and 2 (Per 1 and 2), cryptochrome circadian regulator 1 (Cry), and mainly Rev‐erb‐α (Triqueneaux et al. 2004). Rev‐erb‐α, also known as nuclear receptor subfamily 1 group D member 1 (NR1D1), is one of the two Rev‐erbs proteins in the nuclear receptor family of intracellular transcription factors (Preitner et al. 2002). As a result, Rev‐erb‐α and the nuclear receptors (RORα, β, and γ) compete to activate and repress clock components, completing an integral accessory loop, which supports rhythmicity (Meng et al. 2008; T. K. Sato et al. 2004). The NR1D1 gene is highly conserved across organisms and exhibits oscillatory expression in skeletal muscle. However, when maintained in constant darkness, Rev‐erbα null animals display a significantly reduced circadian period and an abnormal phase‐shifting response to light stimuli (Preitner et al. 2002).

Relevantly, Rev‐erb‐α is part of the accessory molecular rhythm and elicits cell‐specific gene programs that contribute to downstream phenotypes in skeletal muscle. Evidence from cell culture experiments, genetic knockout animals, and pharmacological interventions demonstrates that Rev‐erb‐α controls muscle mass and function as well as tissue remodeling (Boulinguiez et al. 2022; Mayeuf‐Louchart et al. 2017). Rev‐erb‐α can promote regulatory mechanisms that decrease atrophy (Mayeuf‐Louchart et al. 2017) and coordinate autophagy pathways in skeletal muscle (Woldt et al. 2013), while improving calcium homeostasis and regenerative process (Boulinguiez et al. 2022). Although Rev‐erb‐α may exhibit distinct mRNA levels across myogenic steps, optimal Rev‐erb‐α levels improve myofiber structure and nuclear concentration post‐injury (Welch et al. 2017). Concomitantly, Rev‐erb‐α can affect the biosynthesis of its endogenous ligand, heme, which intricately modulates metabolic pathways and promotes energy expenditure (Boulinguiez et al. 2022). Accumulating evidence indicates that Rev‐erb‐α positively affects lipid metabolism, calcium homeostasis (Boulinguiez et al. 2022), energy balance, mitochondrial biogenesis, and oxidative capacity in skeletal muscle, which are essential for tissue integrity and health (Woldt et al. 2013). Thus, this specific nuclear receptor can link muscle metabolism to growth and show how muscle adapts to stress and exercise (Mansingh and Handschin 2022).

Exercise training serves as a powerful zeitgeber (time cue) that can modify the amplitude and phase of the circadian rhythm in skeletal muscle, thereby changing the clock gene signature (Saner et al. 2018; Schroder and Esser 2013). In our laboratory, we previously demonstrated that a single bout of endurance exercise promotes early *Nr1d1* activation, resulting in an upregulation of *Nr1d1* mRNA levels in the soleus muscle of mice (Rovina et al. 2021). Furthermore, Rev‐erb‐α protein content is responsive to various acute exercise modalities and intensities (aerobic, resistance, and interval training) in humans and rodents (Popov et al. 2018). However, the transcription of most of these clock genes, including *Nr1d1* expression, demonstrates bimodal regulation, which starts with repression immediately after acute exercise and subsequently undergoes upregulation, returning to baseline (Popov et al. 2018; Van der Stede et al. 2024). On the other hand, chronic free‐running wheel and treadmill aerobic training can increase the transcriptional and translational levels of Rev‐erb‐α in the skeletal muscle of adult and prematurely aging mice (Vitorino et al. 2025; Woldt et al. 2013; Yao et al. 2024). Elsewhere, higher levels of physical activity can increase the amplitude of Rev‐erb‐α over 24 h in human myotubes (Hansen et al. 2016). Global Rev‐erb‐α knockout in rodents leads to reduced muscle mass, diminished force generation (Boulinguiez et al. 2022), and impaired endurance during prolonged exercise. These alterations were accompanied by lower maximal oxygen consumption (VO_2_max) and diminished mitochondrial respiratory capacity (Woldt et al. 2013), reinforcing the importance of this molecule for metabolic benefits.

Previous reviews have extensively focused on the role of exercise in modulating the primary arm of the core loop (CLOCK‐BMAL1 axis) (Mansingh and Handschin 2022; Martin and Esser 2022; Su and Xiang 2025), whereas the involvement of the accessory loop in skeletal muscle remains underexplored. Understanding how Rev‐erb‐α orchestrates the physiological adaptations of skeletal muscle to exercise remains a critical knowledge gap, with potential implications for muscle health and metabolic regulation. Thus, this integrative review clarifies the emerging hypothesis that Rev‐erb‐α acts as a central hub mediating adaptation to exercise. First, we clarify the complex molecular mechanisms underlying Rev‐erb‐α activation and its effects on myogenesis, metabolism, and autophagy. Next, we discuss frontier‐of‐knowledge findings on the time‐course of Rev‐erb‐α molecular signatures and the distinct acute and chronic exercise responses. Furthermore, we critically analyze the effects of different exercise modalities on the Rev‐erb‐α signaling pathways in rodents and humans. It is valuable to interpret recent advances that used primary and secondary cell culture, knockout mouse models and pharmacological agents to elucidate the pleiotropic impacts of Rev‐erb‐α and its biological significance. The contemporary view of skeletal muscle's multifaceted roles will contribute to understanding the interplay among the circadian cycle, metabolic health, and cellular homeostasis. Thus, elucidating the clinical and translational roles of Rev‐erb‐α in skeletal muscle may enable circadian‐based precision medicine and reveal chronotherapeutic targets in exercise physiology.

## 2 Complex Molecular Mechanisms Regulating Rev‐erb‐α Stability and Activation: Transcriptional, Translational, and Post‐Translational Responses

NR1D1 (Rev‐erb‐α) and NR1D2 (Rev‐erb‐β) are paralogous molecules that share approximately 71% amino acid identity, and each contains a DNA‐binding domain (DBD) composed of two C4 zinc‐finger motifs (Ikeda et al. 2019). Unlike classical nuclear receptors, Rev‐erb‐α and Rev‐erb‐β lack the AF‐2 helix required for coactivator recruitment and therefore function as constitutive transcriptional repressors within a negative feedback loop. NR1D2 shares circadian and repressive functions with NR1D1 but has subtler, context‐dependent, and partially redundant roles in skeletal muscle metabolism, indicating functional overlap (Andrew C Liu et al. 2008). However, NR1D2 exhibits gene‐specific effects, including modulation of lipid pathways, indicating selective lipogenic regulation (Amador et al. 2018; Ramakrishnan et al. 2005). Despite similarities, NR1D1 mainly influences skeletal muscle remodeling, mitochondrial phenotype, and autophagy, thereby contributing to muscle morphology and performance (Woldt et al. 2013).

Intricate transcriptional, translational, and post‐translational events contribute to the localization, stability, and function of Rev‐erb‐α. The CLOCK:BMAL1 heterodimer binds E‐box elements in the NR1D1 promoter and rhythmically activates NR1D1 transcription (Zhang‐Sun et al. 2023), with expression typically peaking during the daytime in skeletal muscle. In turn, Rev‐erb‐α binds ROR response elements (ROREs) in the promoters of target genes, including BMAL1, and represses their transcription. This reciprocal regulation forms a feedback loop that is crucial for maintaining circadian oscillations. At ROREs, Rev‐erb‐α competes with RORα, which activates transcription at the same response elements. The balance between these opposing activities helps fine‐tune the amplitude and timing of circadian target‐gene expression. Rev‐erb‐α activity is also modulated by metabolic cues, including its endogenous ligand, heme (Lazar 2016). Heme binding enhances the transcriptional repressor activity of Rev‐erb‐α by stabilizing its interaction with the NCoR/HDAC3 corepressor complex, thereby promoting histone deacetylation and reinforcing the repression of target‐gene transcription (Lazar 2016; Yin et al. 2010).

Specifically, Rev‐erb‐α can be phosphorylated at specific residues, leading to different biological outcomes. Phosphorylation by GSK‐3β at serine 55 and 59 stabilizes the protein (Yin et al. 2006). This phosphorylation event is associated with enhanced transcriptional repressor activity, leading to activation, nuclear translocation, and increased transcription. This stabilization is crucial for maintaining the circadian rhythm, particularly for the RORα repression and RORE‐containing genes. On the other hand, CK1ε phosphorylates the N‐terminal regions, thereby affecting the protein's ability to accumulate in the cytoplasm. Also, phosphorylation by CDK1 can target Rev‐erb‐α for proteasomal degradation via the F‐box protein FBXW (Zhao et al. 2016). This process is linked to regulating the amplitude of the circadian clock and maintaining whole‐body lipid and glucose homeostasis.

Heat shock proteins (HSPs) play a crucial role in maintaining the proteostasis of Rev‐erb‐α by facilitating its proper folding, functionality, and timely degradation. In addition, HSPs serve as scaffolding molecules that coordinate the assembly and interaction of various proteins. Research has shown that Rev‐erb‐α interacts with HSP90, influencing the stability of the glucocorticoid receptor (Okabe et al. 2016). As a result, these nuclear receptors interact, altering each other's transcriptional activity and causing glucocorticoid receptor target genes to be expressed in a time‐dependent manner (Okabe et al. 2016).

Ubiquitin ligases that regulate protein stability drive Rev‐erb‐α degradation. For example, Siah2 overexpression destabilizes Rev‐erb‐α, whereas Siah2 depletion stabilizes the endogenous protein, delaying its degradation and lengthening the circadian period (DeBruyne et al. 2015). Similarly, Spsb4‐mediated Rev‐erb‐α degradation could be blocked by proteasome inhibition with MG132 in a robust cell‐based degradation assay, thereby influencing protein turnover (Mekbib et al. 2019). More recently, some authors have suggested a lysine‐independent ubiquitination mechanism, implying N‐terminal ubiquitination. This process is mediated by a bifunctional degradation control sequence at the protein's N‐terminus. Exactly, deleting the first nine amino acids of Rev‐erb‐α accelerates its degradation.

Additionally, a region within the first 25 amino acids is critical for interaction with the E3 ubiquitin ligase SPSB4, which mediates this degradation (Mekbib et al. 2019). Fbxl3‐deficient mice exhibit increased Rev‐erb‐α expression, suggesting that FBXL3, as part of the SCF ubiquitin ligase complex, governs Rev‐erb‐α turnover through indirect mechanisms (Shi et al. 2013). However, SUMOylation of Rev‐erb‐α can trigger its ubiquitination, decreasing protein levels. This reduction promotes heightened glycolysis and disrupts normal inflammatory pathways during gastric cancer progression (Ke et al. 2022). In HEK293T cells, Interleukin‐1 (IL‐1) stimulates Rev‐erb‐α Sumoylation, accelerating its protein breakdown (Pariollaud et al. 2018).

Although not tested in C2C12 or muscle fiber, DNA hypermethylation of CpG dinucleotides (CpG islands) can lead to transcriptional and translational repression of Rev‐erbα in microglia and bone marrow‐derived macrophages (Nam et al. 2022). Mechanistically, overactivation of the PI3K/AKT1 pathway triggers upregulation of DNA methyltransferase 3A (DNMT3a) (Figure 1), which adds methyl groups to DNA (Nam et al. 2022), reducing Rev‐erb‐α expression in a knock‐in mouse model of familial Alzheimer's disease (*Psen2* ^N141I/+^ mice). Pharmacological restoration of Rev‐erb‐α levels reversed the hyperinflammatory phenotype and associated cognitive deficits in vivo, establishing Rev‐erb‐α downregulation as a mechanistic link between circadian gene dysregulation and immune dysfunction (Nam et al. 2022).

**Figure 1:**
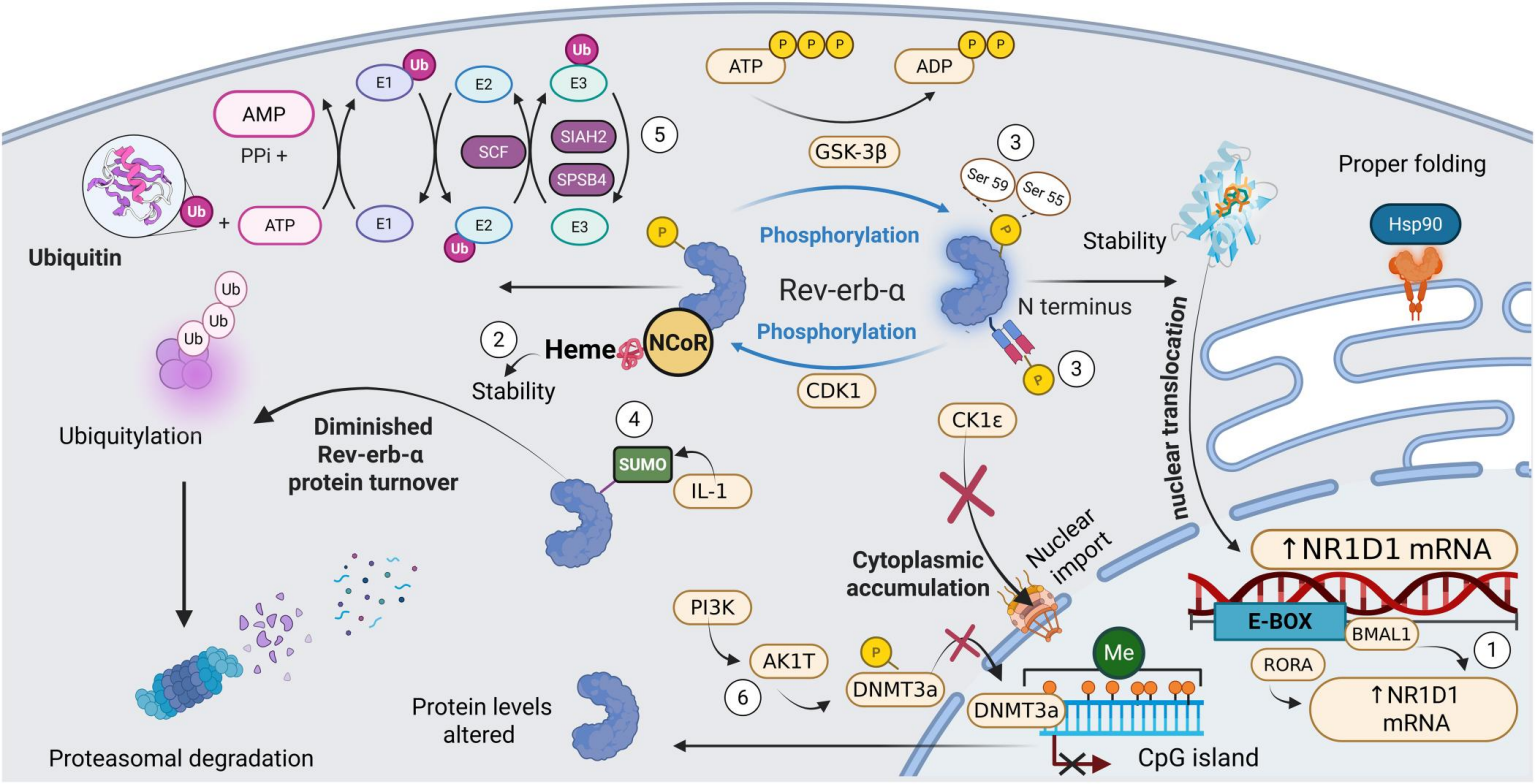
Transcriptional, translational, and post‐translational mechanisms involved in Rev‐erb‐α. (1) Bmal1 and ROR are the primary mechanisms governing Rev‐erb‐α transcriptional levels in the feedback loop. (2) Heme binding enhances Rev‐erb‐α activity by stabilizing its interaction with the NCoR/HDAC3 corepressor complex, reinforcing transcriptional repression of target genes by histone deacetylation. (3) Phosphorylation of serine residues 55 and 59, as well as the N‐terminal region, exerts opposing effects on the protein's localization and activity. (4) IL‐1‐induced sumoylation of Rev‐erb‐α triggers ubiquitination, proteasomal degradation, and protein turnover. (5) Siah2, Spsb4, and Scf mediate the ubiquitination and proteasomal degradation of Rev‐erb‐α. (6) Overactivation of the PI3K/AKT1 pathway leads to Rev‐erb‐α methylation by upregulation of DNMT3a. AKT1, serine/threonine‐protein kinase AKT1; BMAL1, brain and muscle ARNT‐like protein 1; DNMT3A, DNA methyltransferase 3A; HDAC3, histone deacetylase 3; IL‐1, interleukin 1; NCoR, nuclear receptor corepressor; PI3K, phosphatidylinositol 3‐kinase; ROR, retinoic acid receptor‐related orphan receptor; SCF, SKP1–Cullin–F‐box protein complex; SIAH2, seven in absentia homolog 2; SPSB4, SPRY domain‐containing SOCS box protein 4.Biorender® web‐based software was used to create the figure.

## 3 The Dual Role of Rev‐erb‐α in the Myogenesis Process, Skeletal Muscle Structure, and Tissue Remodeling

To characterize the effects of Rev‐erb‐α on skeletal muscle mass, Mayeuf‐Louchart et al. (2017) combined in vitro and in vivo approaches to investigate the role of this receptor in muscle growth. Rev‐erb‐α‐deficient mice exhibited lower tibialis anterior muscle mass and cross‐sectional area (CSA), along with a shift in fiber‐size distribution toward smaller diameters, compared with wild‐type mice. Consistent with the in vivo findings, Rev‐erb‐α ‐deficient C2C12 myotubes exhibited reduced diameters. Global Rev‐erb‐α deficiency also coordinately increased the expression of the atrophy‐related genes Fbxo32 (Atrogin‐1) and Trim63 (MuRF1) in mouse skeletal muscle. Conversely, Rev‐erb‐α overexpression and pharmacological activation with SR9009 in C2C12 cells counteracted dexamethasone‐induced atrophic changes (Mayeuf‐Louchart et al. 2017).

A prior study showed that Rev‐erb‐α promotes myoblast differentiation by activating mTORC1, as overexpression of Rev‐erb‐α decreases *Tsc1* mRNA and protein levels while activating TORC1 in myotubes. In contrast, knockdown induces the opposite effects (Dadon‐Freiberg et al. 2020). Concomitantly, siRev‐erb‐α C2C12 myotubes displayed lower protein levels of MyoD, MHC, and p706K. Furthermore, the absence of Rev‐erb‐α in zebrafish leads to altered muscle growth and reduced muscle function, resulting in premature sarcopenia in adult animals. The authors discovered that nocturnal catabolism is driven by the changing balance of ROR and Rev‐erb‐α transcription factors from the muscle clock stabilization loop, which suppresses TORC1 activity (Kelu and Hughes 2025). Thus, the results suggest that Rev‐erb‐α normally suppresses muscle wasting.

In addition, the peak of *Nr1d1* mRNA levels and the rhythmicity of metabolic genes were decreased in denervated muscle (gastrocnemius), which might have caused a phase advance of Arntl mRNA levels in the sciatic denervation model (Nakao et al. 2015). Also, transcriptomic analysis confirmed lower *Nr1d1* levels in different skeletal muscles of mice exposed to the denervation model, dexamethasone, spaceflight, and bedrest (Malhan et al. 2023). Consequently, the dysregulated *Nr1d1* expression induced by atrophy impairs the capacity of skeletal muscle.

In contrast to previous studies, Chatterjee et al. (2019) highlighted that Rev‐erb‐α can suppress myogenesis. It was demonstrated that primary myoblasts deficient in Rev‐erb‐α exhibit improved proliferation and myogenic differentiation, as evidenced by elevated expression of myogenic regulatory factors (MRFs), such as Myf5, Myod, and myogenin during the differentiation compared with WT cells. Furthermore, pharmacological activation of Rev‐erb‐α reduces MRFs, whereas its inhibition by an antagonist (SR8278) increases MRFs expression levels. Remarkably, the global Rev‐erb‐α deletion in mice enhances satellite cell proliferative expansion and accelerates regenerative progression following muscle injury (Chatterjee et al. 2019). However, no significant effect of Rev‐erb‐α ablation on muscle growth was evident under normal conditions. These apparently divergent findings may reflect stage‐ and model‐dependent functions of Rev‐erb‐α, including regulation of proliferation and differentiation in myogenic progenitors and distinct metabolic and proteostatic functions in differentiated muscle. Although the in vitro findings support a cell‐autonomous role for Rev‐erb‐α, a global knockout model means contributions from non‐muscle tissues to the in vivo phenotype cannot be excluded. Also, a potential limitation is that primary myoblasts isolated from multiple skeletal muscles may represent a heterogeneous cell population with distinct fiber‐type composition and myogenic programs, increasing biological variability and obscuring muscle‐specific effects.

In this context, Welch et al. (2017) investigated the effects of pharmacological Rev‐erb‐α antagonism with SR8278 in C57BL/10ScSn‐Dmd^mdx^/J (mdx) mice, a model of Duchenne muscular dystrophy. SR8278 treatment improved muscle function and reduced skeletal muscle fibrosis (Welch et al. 2017). These effects were accompanied by activation of Wnt and Notch signaling, two interconnected pathways that jointly regulate satellite‐cell activation, self‐renewal, proliferation, differentiation, and fusion during muscle regeneration (Welch et al. 2017). A deeper understanding of the mechanistic role of Rev‐erb‐α in myogenic progenitor‐cell behavior may therefore reveal novel strategies to enhance regenerative capacity and preserve muscle mass. Nevertheless, findings obtained using pharmacological modulators should be interpreted cautiously because the receptor specificity, binding affinities, and potential off‐target effects of currently available Rev‐erb‐α agonists and antagonists remain incompletely characterized.

The conflicting findings reported in the literature may be due to Rev‐erb‐α‘s dual roles in myogenesis and tissue remodeling. The involvement of Rev‐erb‐α in myocytes was initially recognized in cultured C2C12 myoblasts, in which Rev‐erb‐α suppresses gene expression associated with cell differentiation (Downes et al. 1995). However, Welch et al. (2017b) found that Rev‐erb‐α may exhibit distinct expression patterns in proliferating and differentiated muscle cells because its promoter occupancy (NF‐Y complex at CCAAT) differs across developmental stages. Initially, the authors observed an increase in Rev‐erb‐α activation during the proliferative phase, but a significant decline during myoblast differentiation. After these processes, Rev‐erb‐α activity was restored in the differentiated muscle. In addition, the authors found that two distinct Rev‐erbα genotypes (Rev‐erbα^−/−^ and Rev‐erbα^+/−^) had larger muscle CSAs than wild‐type mice. Compared to wild‐type controls, heterozygous mice exhibited enhanced regeneration, rapidly restoring myofiber architecture, increased myonuclear density, and enlarged fiber CSA following injury (Welch et al. 2017). These observations suggest that diminished Rev‐erb‐α expression accelerates muscle repair following injury. In contrast, complete loss of Rev‐erb‐α expression can negatively affect muscle regeneration, suggesting a dose threshold for Rev‐erb‐α in regulating muscle homeostasis.

More recently, Boulinguiez et al. (2022) found that the capacity for sarco/endoplasmic reticulum calcium ATPase‐dependent calcium uptake into the reticulum was impaired in mice lacking *Nr1d1*. Thus, pharmacological activation of NR1D1 with the SR9009 agonist may confer therapeutic benefit by enhancing calcium release in muscle cells from patients with Duchenne muscular dystrophy. Also, SR9009 improved the myopathy phenotype in mdx/Utr^+/–^ mice by upregulating SERCA activity, while repressing myoregulin in the gastrocnemius muscle. Furthermore, fibrosis markers, such as *Col1a2* and *Pdgfra* mRNA levels, Sirius red staining (collagen content), and hydroxyproline amount, were also reduced by SR9009 treatment, contributing to improved muscle contraction. Regarding practical applications, NR1D1 emerges as a novel regulator of sarcoplasmic reticulum calcium homeostasis, making it a promising drug target for skeletal muscle disorders characterized by disrupted calcium equilibrium, as observed in many myopathic diseases.

Intriguingly, lower body weight and smaller Type I fibers in the soleus of Rev‐erbα^−/−^ animals compared to wild‐type controls have been observed, suggesting a mild general growth retardation (Pircher et al. 2005). These results agree with higher Rev‐erb‐α levels in red muscles, which are rich in mitochondria and aerobic metabolism and demonstrate prolonged energy mobilization in response to exercise (Woldt et al. 2013). The synthesis of Rev‐erb‐α effects on biological processes governing muscle mass, function and biology is reported in Figure 2.

**Figure 2:**
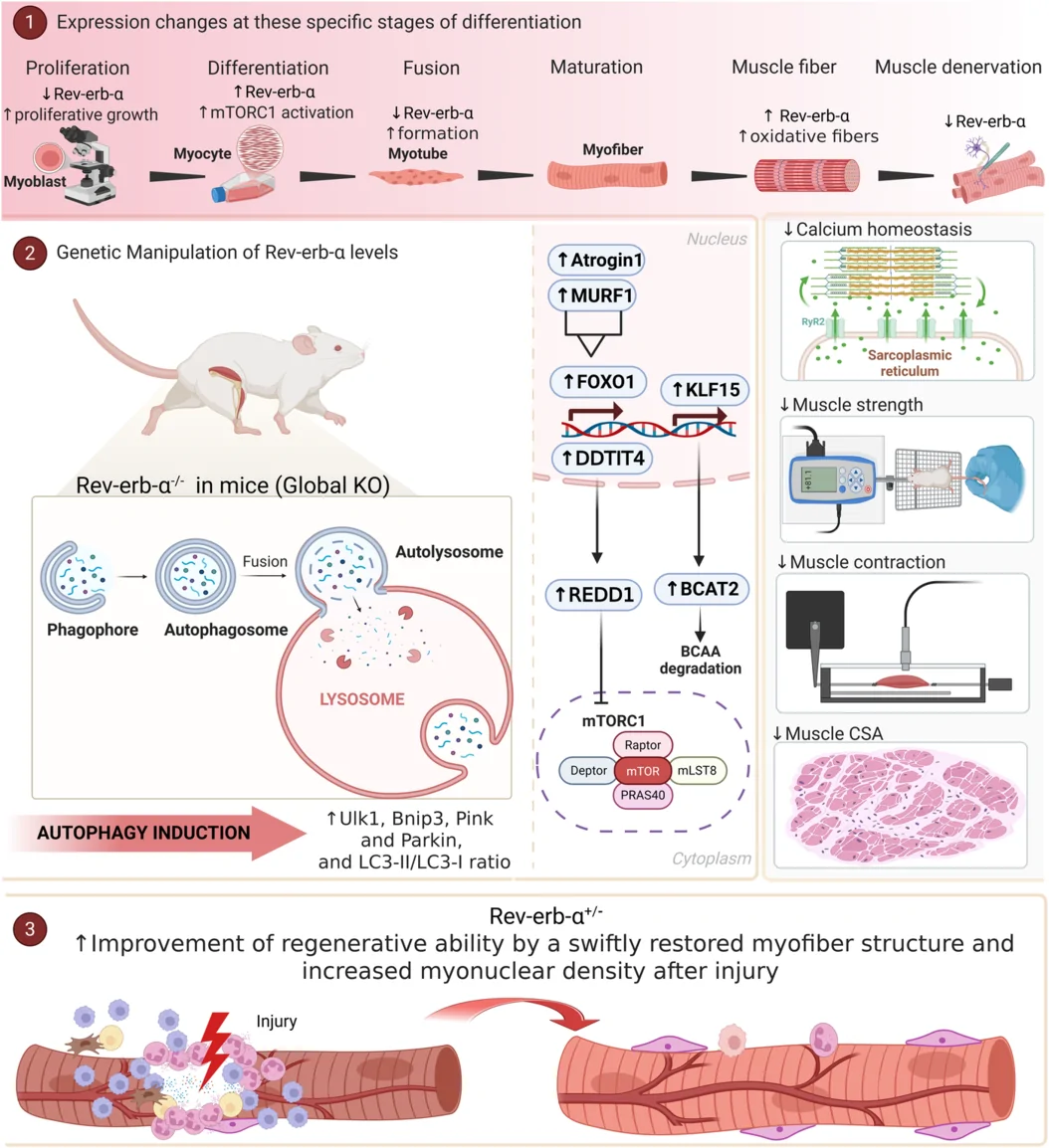
Schematic description of the Rev‐erb‐α effects on skeletal muscle development, function and growth. (1) Rev‐erb‐α displays distinct levels in the myogenesis process, oxidative fibers, and the denervation model. (2) In the full knockout mice (global KO), the loss of Rev‐erb‐α increased autophagy targets (Ulk1, Bnip3, Pink, and Parkin), atrophy pathways (Foxo1, Murf11, and Ddtit4) and BCAA degradation (Klf15 and Bcat2) in a coordinated manner, leading to decreased muscle cross‐sectional area (CSA), impaired muscle contraction, muscle strength and disrupted calcium homeostasis in skeletal muscle. (3) Heterozygous animals (Rev‐erbα^+/−^) showed an improved regenerative ability with nuclear concentration and enlarged muscle fiber cross‐sectional area (CSA) post‐injury compared to wild‐type mice. (REV‐ERBα) nuclear receptor subfamily 1 group D member 1; Atrogin‐1, muscle atrophy F‐box protein; BCAA, branched‐chain amino acids; BCAT2, branched‐chain amino acid aminotransferase 2; BNIP3, BCL2‐interacting protein 3; CSA, cross‐sectional area; DDIT4, DNA damage‐inducible transcript 4; DEPTOR, DEP domain‐containing mTOR‐interacting protein; FOXO1, forkhead box O1; KLF15, Krüppel‐like factor 15; KO, knockout; LC3, microtubule‐associated protein 1 light chain 3; mLST8, mammalian lethal with SEC. 13 protein 8; mTOR, mechanistic target of rapamycin; mTORC1, mechanistic target of rapamycin complex 1; MuRF1, muscle RING‐finger protein 1; Parkin, parkin RBR E3 ubiquitin‐protein ligase; PINK1, PTEN‐induced kinase 1; PRAS40, proline‐rich AKT substrate of 40 kDa; RAPTOR, regulatory‐associated protein of mTOR; REDD1, regulated in development and DNA damage response 1; RyR2, ryanodine receptor 2; ULK1, Unc‐51‐like autophagy‐activating kinase 1. Biorender® web‐based software was used to create the figure.

## 4 Rev‐erb‐α Directly Inhibits Autophagy Activation in Skeletal Muscle

The circadian clock regulates the timing and activity of autophagy, synchronizing cellular self‐degradation and recycling with metabolic demands, a process critical for maintaining muscle mass, function, and homeostasis (Kelu and Hughes 2025). Recent studies indicate that the molecular circadian clock directly orchestrates rhythmic autophagy through the interplay of the regulators RORα/γ and NR1D1 within the muscle peripheral clock (Kelu and Hughes 2025).

Interestingly, overexpressing NR1D1 in muscle fibers may promote hypertrophy by dampening autophagic activity (Kelu and Hughes 2025). Pharmacologically, the Rev‐erb‐α agonist SR9011 suppresses night‐time autophagy flux, while the antagonist SR8278 enhances it. These effects are likely driven by changes in the phosphorylation status of Ulk1 at Ser757 and RpS6 at Ser240/244, both of which are up‐ or downregulated depending on whether an agonist or an antagonist is applied (Kelu and Hughes 2025).

Consequently, Rev‐erb‐α modulates autophagy by downregulating a set of genes critical for vesicle expansion, autophagosome assembly, and lysosomal degradation. In mice lacking Rev‐erb‐α (Rev‐erbα^−/−^), skeletal muscle displayed elevated levels of mRNA levels of *Ulk1* and *Beclin1*, as well as Bnip3 protein levels, along with lysosomal enzymes such as cathepsin L and ATPase6v1b2, compared to their male wild‐type counterparts (Rev‐erbα^+/+^) (Woldt et al. 2013). Functional validation of increased autophagy was provided by experiments using lysosome inhibitors (bafilomycin and ammonium chloride) in C2C12 cells engineered to overexpress Rev‐erb‐α (Woldt et al. 2013). These cells exhibited elevated LC3 protein levels, suggesting that autophagic flux may be hampered. To delve deeper into the underlying mechanisms, the researchers found that Rev‐erb‐α binding sites were significantly enriched within the regulatory regions of multiple autophagy‐related genes in C2C12 myotubes, as shown by chromatin immunoprecipitation sequencing (ChIP‐seq) (Woldt et al. 2013). This binding correlated with reduced histone acetylation at the H3K27 and H3K9 marks in those genomic regions.

It was similarly investigated how *Nr1d1* was expressed in the muscles of tilapia treated with GSK4112, a Rev‐erb‐α agonist (P. Wu et al. 2020). Their findings showed that *Nr1d1* levels were markedly elevated in the muscles of treated fish. In contrast, key autophagy‐related genes, including *Atg4c*, *Atp6v1d*, *Map1lc3a*, and *Ulk1a*, were significantly reduced compared to untreated controls. These results confirm a major role for NR1D1 in downregulating autophagy‐associated gene expression in skeletal muscle.

During exercise, many of the autophagy‐related genes analyzed showed reduced transcription, coinciding with a drop in Rev‐erb‐α expression. Notably, there was a moderate inverse relationship between Rev‐erb‐α and certain autophagy. In particular, *Map1lc3b* was significantly downregulated in the gastrocnemius muscle following acute aerobic exercise, supporting the repressive activity of Rev‐erb‐α on autophagy‐related genes (da Rocha et al. 2022). In addition to its transcriptional roles, Rev‐erb‐α modulates Parkin expression, a critical mediator of mitophagy (Woldt et al. 2013). By controlling Parkin levels, Rev‐erb‐α promotes mitochondrial biogenesis, enforces quality control, and facilitates the clearance of dysfunctional organelles. The specific cellular activities and targets of Rev‐erb‐α are summarized in Figure 3.

**Figure 3:**
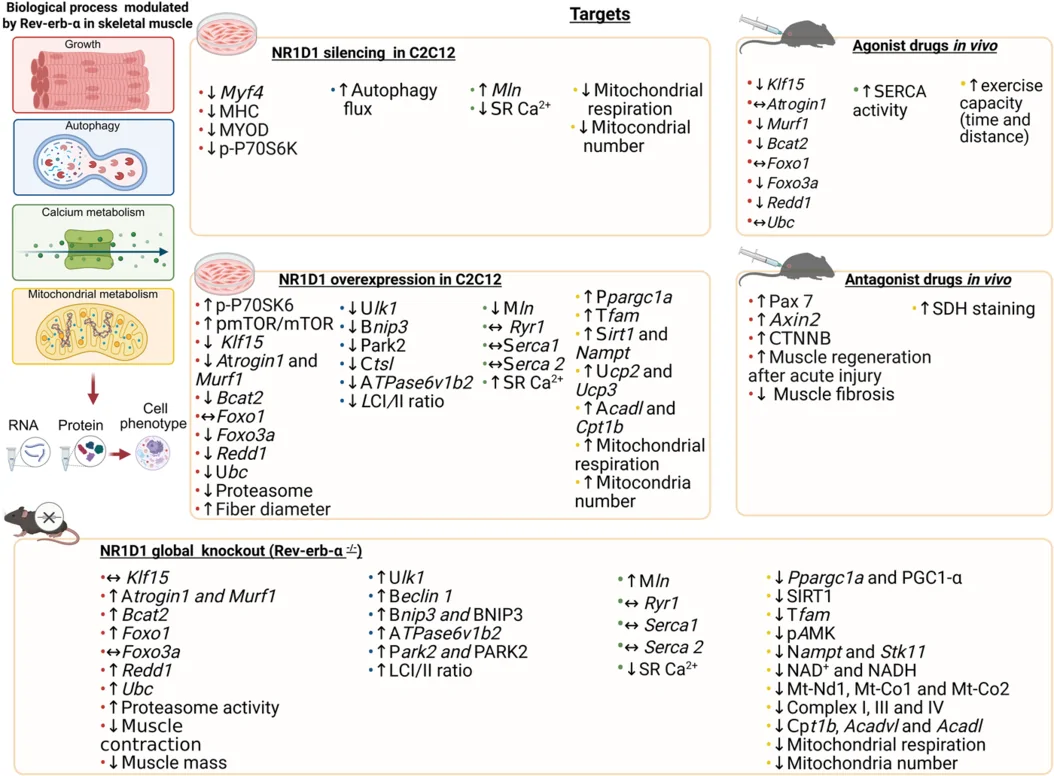
The main biological processes, targets, and phenotypes regulated by Rev‐erb‐α in cell culture models, pharmacological agents, and full mice knockout. Red: muscle growth, blue: autophagy, green: calcium metabolism and yellow: mitochondrial metabolism. *Nr1d1* silencing and overexpression in C2C12, Agonist and antagonist drugs in wild‐type mice (in vivo approach) and NR1D1 global knockout mice (Rev‐erb‐α^−/−^). Changes in mRNA levels, protein content, and cell phenotype were reported for each condition. (↓) Downregulation (↑), upregulation and (↔) no change. ACADL, long‐chain acyl‐CoA dehydrogenase; ACADVL, very‐long‐chain acyl‐CoA dehydrogenase; AMPK, AMP‐activated protein kinase; ATP6V1B2, V‐type proton ATPase subunit B2; AXIN2, axis inhibition protein 2; BCAA, branched‐chain amino acids; BCAT2, branched‐chain amino acid aminotransferase 2; BECN1, beclin 1; BNIP3, BCL2‐interacting protein 3; C2C12, murine skeletal muscle myoblast cell line; CPT1B, carnitine palmitoyltransferase 1B; CTNNB1, catenin beta 1; CTSL, cathepsin L; FOXO1, forkhead box O1; FOXO3A, forkhead box O3A; KLF15, Krüppel‐like factor 15; KO, knockout; LC3, microtubule‐associated protein 1 light chain 3; MHC, myosin heavy chain; MLN, myoregulin; mTOR, mechanistic target of rapamycin; MuRF1, muscle RING‐finger protein 1; MYF4, myogenic factor 4; MYOD, myogenic differentiation 1; NAD^+^, oxidized nicotinamide adenine dinucleotide; NADH, reduced nicotinamide adenine dinucleotide; NAMPT, nicotinamide phosphoribosyltransferase; NR1D1/REV‐ERBα, nuclear receptor subfamily 1 group D member 1; p70S6K, 70‐kDa ribosomal protein S6 kinase; PARK2, parkin RBR E3 ubiquitin‐protein ligase; PAX7, paired box 7; PGC‐1α/PPARGC1A, peroxisome proliferator‐activated receptor gamma coactivator 1‐alpha; REDD1, regulated in development and DNA damage response 1; RYR1, ryanodine receptor 1; SDH, succinate dehydrogenase; SERCA, sarco/endoplasmic reticulum calcium ATPase; SIRT1, sirtuin 1; SR, sarcoplasmic reticulum; STK11, serine/threonine kinase 11; TFAM, mitochondrial transcription factor A; UBC, ubiquitin C; UCP2, uncoupling protein 2; UCP3, uncoupling protein 3. Symbols: (p‐) phosphorylated; (Ca^2+^) calcium ion; (↑) increase; (↓) decrease; (↔) no significant change; and (−/−) homozygous knockout. Biorender® web‐based software was used to create the figure.

## 5 Rev‐erb‐α Integrates Primary Metabolism and Mitochondrial Function in Skeletal Muscle

There is growing evidence that the nuclear receptor Rev‐erb‐α regulates energy metabolism in skeletal muscle, including fat deposition, gluconeogenesis (Welch et al. 2020), and cholesterol homeostasis (Zhang‐Sun et al. 2023). It is also necessary to balance carbohydrate and lipid utilization daily, as this affects primary metabolism and bioenergetics (Carter et al. 2016). Molecularly, heme binds directly to Rev‐erb‐α in a 1:1 stoichiometry (Raghuram et al. 2007; Yin et al. 2007) and, conversely, Rev‐erb‐α regulates heme synthesis, which is intricately linked to energy homeostasis (Yin et al. 2010). This crosstalk modulates metabolic flexibility by altering the activity and expression of metabolic enzymes and transcription factors through heme binding.

Delezie et al. (2012) showed that the lipogenic genes are disrupted in Rev‐erbα^−/−^ animals exposed to a high‐fat diet in the rectus femoris skeletal muscle. These mice displayed downregulation of *Glut4* mRNA levels, while lipoprotein lipase (LPL) was constitutively upregulated, contributing to fat storage. Furthermore, the knockout mice exhibit slight hyperglycemia and are more susceptible to HFD‐induced obesity. This feature could be due to a shift from glucose to fatty acid utilization for energy supply, at least during the resting period and 24 h of food withdrawal (Delezie et al. 2012). Thus, loss of Rev‐erb‐α can dysregulate the molecular control of energy homeostasis, contributing to the development of obesity‐related disorders.

Mechanistically, elevated Rev‐erb‐α expression leads to recruitment of HDAC3 to the genome, which prepares the muscle to utilize glucose in cooperation with systemic assimilation of carbohydrates into protein or lipid during the feeding cycle (Hong et al. 2017). Consequently, removing HDAC3 from the genome reduces glucose absorption and increases amino acid breakdown under normal physiological conditions (Hong et al. 2017). Therefore, numerous genes linked to BCAA catabolism (*Ampd3*, *Bcat3*, *Dbt*, *Glul*, *Shmt1*, and *Ivd*) were upregulated in the quadriceps muscle of Rev‐erb‐α KO mice, further supporting a pivotal role of Rev‐erb‐α in skeletal muscle remodeling (Hong et al. 2017).

In a similar experimental model, *Nr1d1* ^−/−^ mice have not been shown to exhibit changes in glucose clearance or insulin sensitivity compared with wild‐type mice, but do exhibit higher resting blood glucose levels (Welch et al. 2020). Regarding molecular pathways, homozygous mice showed increased expression of the *Hk1* and *Hk2* genes, which encode hexokinase 1 and 2, respectively. This upregulation is commonly observed in various pathological conditions, including low‐grade inflammation, aging, diabetes, and tumor progression (Zapater et al. 2022). On the other hand, heterozygous mice exhibited increased glucose uptake during fasting and elevated basal gluconeogenesis compared with wild‐type and knockout littermates, indicating that glucose metabolism is sensitive to varying levels of Rev‐erb‐α (Welch et al. 2020).

The initial evidence for the beneficial roles of Rev‐erb‐α in mitochondrial adaptations in skeletal muscle was presented 12 years ago. In a classic study, Woldt et al. (2013) found that global Rev‐erb‐α deficiency reduces mitochondrial DNA content, biogenesis, and electron transport chain activity in skeletal muscle. These features were linked to dysregulated Ampk‐Sirt1‐Ppargc1‐α signaling pathway. Also, the researchers observed that Pgc1‐α acetylation is higher in Rev‐erb‐α‐deficient cells, which may contribute to lower mRNA levels of important transcription factors involved in mitochondrial function, including mitochondrial transcription factor A (*Tfam*) and Nuclear respiratory factor 1 (*Nrf1*). Also, the same specific phenotype was observed in isolated fibers and C2C12 cells upon Rev‐erb‐α knockdown. Otherwise, Rev‐erb‐α overexpression increased mitochondrial number, respiratory capacity, and the protein content of complexes I and III, reinforcing the positive effects on mitochondrial homeostasis.

In agreement with previous investigations, Rev‐erb‐α overexpression in C2C12 cells increased gene expression of Ppargc1‐α and Rora, as well as MitoTracker staining (Amador et al. 2018). Additionally, enzymes involved in fatty acid β‐oxidation, such as Uncoupling protein 2 (Ucp2), Uncoupling protein 3 (Ucp3), long‐chain acyl‐CoA dehydrogenase (Acadl), short‐chain acyl‐CoA dehydrogenase (Acads), and carnitine palmitoyltransferase 1b (Cpt1b) were substantially upregulated. In contrast, overexpression downregulates fatty acid synthase (Fasn), stearoyl‐CoA desaturase‐1 (Scd1), and sterol regulatory element‐binding protein 1 (Srebf1), key regulators of lipogenesis (Amador et al. 2018), as shown in Figure 4.

**Figure 4:**
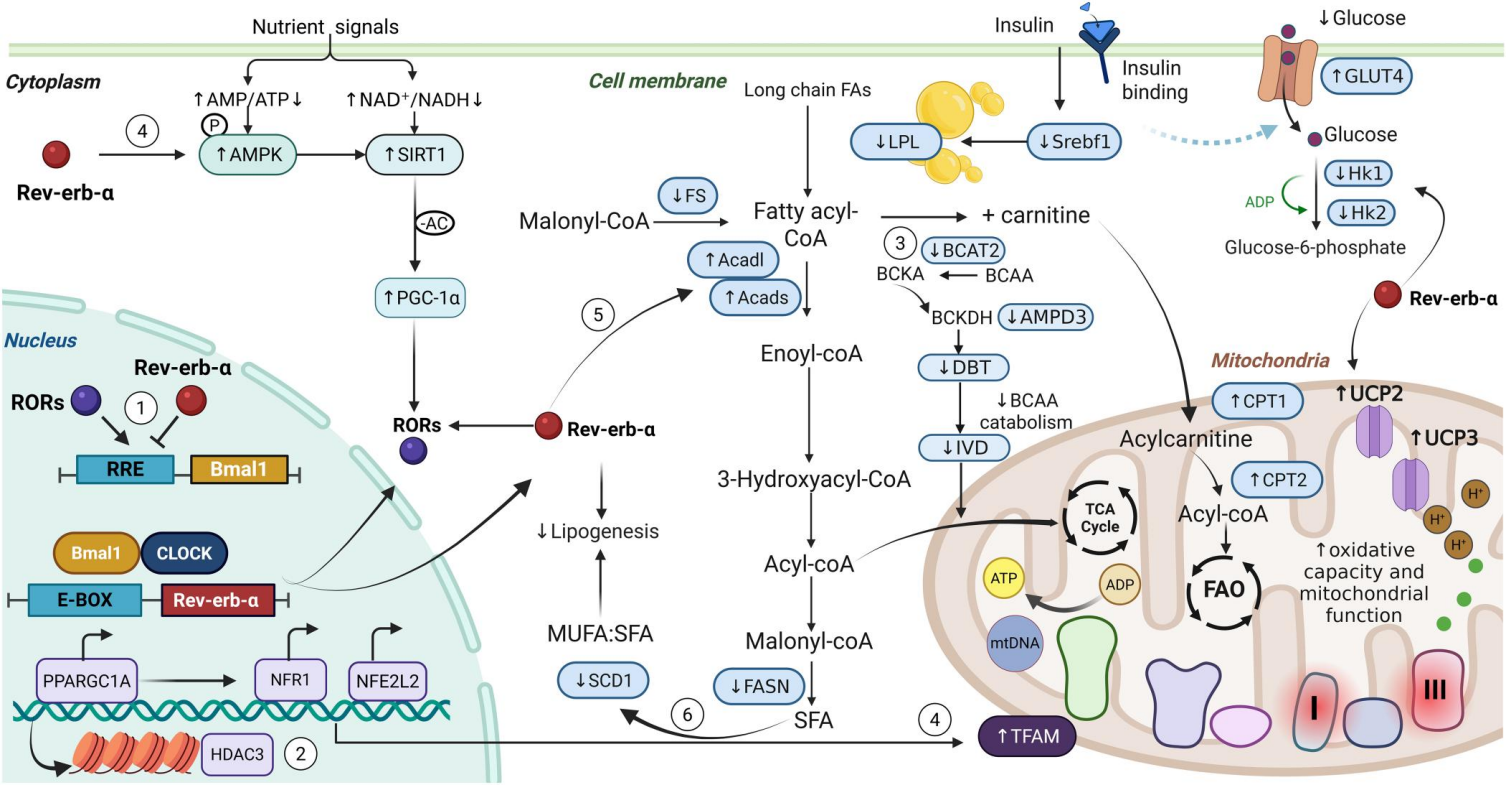
Rev‐erb‐α regulates fuel metabolism and bioenergetics in skeletal muscle. (1) ROR and Rev‐erb‐α compete for ROR‐response elements on the Bmal1 gene, leading to activation or repression of Bmal1 expression. (2) At the molecular level, elevated Rev‐erb‐α levels trigger the recruitment of HDAC3, setting the stage for muscle tissue to efficiently use glucose in coordination with the conversion of carbohydrates into proteins or fats during feeding. (3) The downregulation of genes involved in BCAA catabolism by Rev‐erb‐α highlights its crucial function in remodeling skeletal muscle. (4) Higher Rev‐erb‐α levels upregulate the AMPK‐SIRT1‐PGC‐1α signaling pathway, leading to increased Tfam and Nrf1 protein and mRNA levels, as well as complex I and III protein content, thereby increasing mitochondrial respiratory capacity. (5) Rev‐erb‐α modulates β‐oxidation‐driven generation of reducing equivalents to feed into the electron transport chain by upregulation of *Ucps*, *Acads* and *Cpt1b* gene expression. (6) In contrast, ectopic Rev‐erb‐α overexpression downregulates enzymes involved in lipogenesis, including *Fasn*, *Scd1*, and *Srebf1* mRNA levels. AC, acetylation; ACADL, long‐chain acyl‐CoA dehydrogenase; ACADS, short‐chain acyl‐CoA dehydrogenase; ADP, adenosine diphosphate; AMP, adenosine monophosphate; AMPD3, adenosine monophosphate deaminase 3; AMPK, AMP‐activated protein kinase; ATP, adenosine triphosphate; BCAA, branched‐chain amino acids; BCAT2, branched‐chain amino acid aminotransferase 2; BCKA, branched‐chain α‐keto acids; BCKDH, branched‐chain α‐keto acid dehydrogenase complex; BMAL1, brain and muscle ARNT‐like protein 1; CLOCK, circadian locomotor output cycles kaput; CoA, coenzyme A; CPT1, carnitine palmitoyltransferase 1; CPT2, carnitine palmitoyltransferase 2; DBT, dihydrolipoamide branched‐chain transacylase E2; E‐box, enhancer box; FA, fatty acid; FAO, fatty acid oxidation; FASN, fatty acid synthase; FS, fatty acid synthesis; GLUT4, glucose transporter type 4; HDAC3, histone deacetylase 3; HK1, hexokinase 1; HK2, hexokinase 2; IVD, isovaleryl‐CoA dehydrogenase; LPL, lipoprotein lipase; mtDNA, mitochondrial DNA; MUFA, monounsaturated fatty acids; NAD^+^, oxidized nicotinamide adenine dinucleotide; NADH, reduced nicotinamide adenine dinucleotide; NFE2L2/NRF2, nuclear factor erythroid 2‐related factor 2; NRF1, nuclear respiratory factor 1; PGC‐1α/PPARGC1A, peroxisome proliferator‐activated receptor gamma coactivator 1‐alpha; REV‐ERBα/NR1D1, nuclear receptor subfamily 1 group D member 1; RORs, retinoic acid receptor‐related orphan receptors; (RRE) REV‐ERB response element; (SCD1) stearoyl‐CoA desaturase 1; (SFA) saturated fatty acids; (SIRT1) sirtuin 1; SREBF1, sterol regulatory element‐binding transcription factor 1; TCA, tricarboxylic acid; TFAM, mitochondrial transcription factor A; UCP2, uncoupling protein 2; UCP3, uncoupling protein 3. Symbols: (H^+^) proton; (P) phosphorylation; (I and III) mitochondrial respiratory‐chain complexes I and III; (↑) increase; and (↓) decrease. Biorender® web‐based software was used to create the figure.

In the pharmacological approach, it was found that SR9009 analogs effectively decreased triglyceride, blood glucose, and total cholesterol levels in the circulation of high‐fat diet mice (Li et al. 2024). Testing causality, this agonist prevents weight gain and insulin resistance induced by circadian disruptions by inhibiting adipogenesis (Yang et al. 2025). Furthermore, in a mouse model of non‐alcoholic steatohepatitis (NASH), SR9009 improved liver pathology by reducing fibrosis and suppressing inflammation, suggesting a promising drug target for treating metabolic diseases and dysfunction in vivo models (Griffett et al. 2020). However, many reported benefits require high or frequent dosing, and off‐target effects have not been described.

## 6 Regulation of Rev‐erb‐α by Acute Exercise in Skeletal Muscle

### 6.1 Animal Models

Understanding responses to acute exercise is essential before investigating chronic adaptations, as acute exercise induces immediate physiological changes, such as increased muscle blood flow, oxygen uptake, and metabolic adjustments, that lay the foundation for the more substantial, long‐term adaptations associated with regular training (Raun et al. 2026). These initial responses are critical for activating the molecular signaling and cellular processes that drive improvements in muscle strength, endurance, and metabolic function (Raun et al. 2026).

From this perspective, acute endurance exercise bouts in mice substantially affect the immediate response of core clock genes following muscle fatigue, exhibiting bimodal regulation: initial repression immediately after exercise, followed by subsequent upregulation (Maier et al. 2022). Specifically, *Nr1d1* mRNA levels in the quadriceps muscle of male mice were reduced 3 h after exercise sessions compared with baseline and the sedentary group (Maier et al. 2022). Furthermore, distinct muscle clock gene signatures were observed after daytime versus night‐time wheel‐running training, highlighting oscillations in rest and active phase. Consequently, this suggests that, in theory, there may be an optimal time of day to enhance the *Nr1d1* molecular response.

da Rocha et al. (2022) compared the time‐dependent responses of skeletal muscle Rev‐erb‐α to different acute exercise modalities, including endurance exercise, interval running, and resistance exercise, in the gastrocnemius and soleus muscles of male mice. Across all exercise modalities, *Nr1d1* mRNA levels were higher at 18 h than at 0 and 6 h after exercise. However, endurance exercise (60% of maximal aerobic capacity at 0% inclination for 60 min) and resistance exercise (10 climbs with an external load corresponding to 75% of body weight and 2 min of recovery between repetitions) reduced *Nr1d1* mRNA levels in both muscles at 12 h after exercise compared with the control group. Interval running, performed for 60 min with intensity alternating every 5 min between 40% and 80% of maximal aerobic capacity, resulted in higher *Nr1d1* mRNA levels at 18 h than endurance exercise, indicating a protocol‐dependent response. At the protein level, all exercise modalities reduced nuclear Rev‐erb‐α content in the gastrocnemius immediately after the exercise session.

Curiously, Rovina et al. (2021) observed an earlier *Nr1d1* activation than previous studies. An acute bout of treadmill exercise (60% of the maximum aerobic capacity at 0% inclination for 60 min) elevated *Nr1d1* mRNA levels immediately after the session only in the predominant oxidative skeletal muscle (soleus) of male mice, accompanied by increased expression of atrophy‐related genes. Also, the *Nr1d1* gene in soleus muscle after endurance exercise strongly correlated with *Mtor*, *Ppargc1a*, and *Bcat2*, reinforcing the hypothesis that Rev‐erb‐α may mediate mitochondrial metabolism. Since *Nr1d1* expression is positively associated with mitochondrial function and oxidative capacity in skeletal muscle, we hypothesize that increased expression following acute exercise might enhance mitochondrial efficiency to adapt to energy demands.

Additionally, Rev‐erb‐α plays a key role in regulating the inflammatory response by modulating cytokine production. It suppresses pro‐inflammatory cytokines such as IL‐6, IL‐1β, and TNF‐α, while enhancing IL‐10 expression (Chandra et al. 2013). This dynamic regulation is possibly due to the suppression of toll‐like receptor 4 and the NF‐κB signaling pathway, leading to downregulation of related genes (Guo et al. 2019). Moreover, it affects macrophage polarization by favoring a shift toward the anti‐inflammatory M2 phenotype over the pro‐inflammatory M1 phenotype (Sitaula et al. 2015). Additionally, Pinto et al. (2022) reveal a fascinating interplay between IL‐6 and Rev‐erb‐α, suggesting that their interaction may influence beneficial adaptations triggered by acute physical exercise (treadmill running at 20 m min^−1^, 10 degrees of inclination, for 90 min). The researchers found that *Nr1d1* mRNA levels were downregulated after an acute exhaustive exercise protocol (1 and 3 h post‐Ex) in male wild‐type and IL‐6 KO mice, but the total Rev‐erb‐α protein levels were lower for the IL‐6 KO group at the basal and post‐exercise conditions when compared to wild‐type under the same state (Pinto et al. 2022), suggesting that the IL‐6 cytokine can affect Rev‐erb‐α dynamics.

### 6.2 Human Studies

The heterogeneity of fiber types in skeletal muscle makes it challenging to decipher the molecular signaling events that drive exercise adaptations. Recently, Stede and co‐workers (Van der Stede et al. 2024) established an optimized workflow for transcriptional profiling of individual human muscle fibers collected from vastus lateralis biopsies immediately and 180 min after cessation of high‐intensity aerobic exercise [warm‐up of 10 min at 90% of gas exchange threshold (GET) and seven high‐intensity bouts (3 min) at 100% of respiratory compensation point (RCP)] in trained young adults (male and females). It was observed that genes related to circadian rhythm regulation (PER1, HES1, and NR1D1) showed a common response in slow and fast fibers with downregulation during recovery (3 h post‐exercise). This suggests that exercise serves as a cue to re‐establish biological clocks within muscle fibers. However, the possibility of natural diurnal variations at the 3‐h recovery point cannot be excluded without a non‐exercise control group.

These findings are supported by similar time‐point analyses conducted by Popov et al. (2018), who investigated acute exercise‐induced gene expression in the vastus lateralis of individuals previously adapted to aerobic training, at 4 and 8 h after acute cycling sessions performed at different intensities (70% or 50% of VO_2_max). Curiously, NR1D1 mRNA levels were reduced at both measured time points and positively correlated with exercise intensity in male amateur endurance‐trained. Thus, it makes sense to infer that NR1D1 intrinsic responses may depend on training variables, particularly exercise intensity.

Recently, Edman et al. (2024) investigated details of the 24‐h molecular landscape in response to a bout of resistance exercise (short warm‐up on a cycle ergometer, followed by four sets at 7‐repetition maximum (7RM) load with 2 min of rest using both leg press and leg extension) using RNA sequencing (RNA‐seq) in skeletal muscle biopsy samples from male healthy untrained humans. The authors observed that NR1D1, PER1, and PER2 mRNA levels were lower at the 3 h time point. At 8 h, NR1D1, PER1, PER2, and PER3 remained lower, while ARNTL (also known as BMAL1) was upregulated (Edman et al. 2024). Thus, acute resistance exercise tended to blunt their excursions and increase clock gene variability compared to sedentary controls. Furthermore, acute resistance exercise modulates the highly interconnected molecular clock targets, each of which influences and synchronizes with the others.

Although electrical stimulation (30 min at 1 Hz, 2 ms pulse duration, 30 V) in C2C12 myotubes did not affect NR1D1 gene expression in an ex vivo model, downregulation of NR1D1 in the vastus lateralis muscle of young men was observed after a 1‐h acute exercise bout (15 min at 80% VO_2_max on a bicycle ergometer) (Small et al. 2020). Similarly, NR1D1 was downregulated in the desuse model (7 days of unilateral lower‐limb suspension) after 2 h post‐acute exercise (4 sets of 10 repetitions at 70% of 1RM, with a 2‐min rest period between sets) in the skeletal muscle of middle‐aged adults (males and females) (Von Ruff et al. 2025). This change may indicate an adaptive response to altered physiological demands resulting from inactivity. These results corroborate findings in animal models and align with existing evidence that NR1D1 is diminished in human skeletal muscle within 1–6 h, highlighting significant circadian modulation that may influence muscle adaptations. Table 1 presents detailed results for different acute exercise protocols on NR1D1 in rodents and humans.

**Table 1:** Effects of acute exercise on Rev‐erb‐α signature modulation in rodents and humans.

Reference	Experimental model and condition	Sex	Age	Exercise dose (frequency, duration, intensity)	Tissue and time analyses	Skeletal muscle phenotype and metabolic effects	Rev‐erb‐α detection assay and findings	Conclusions on Rev‐erb‐α function
Maier et al. (2022)	C57BL/6 mice	Male	8 weeks of age	Acute treadmill exercise until exhaustion (3 min at 8 m min^−1^, increasing treadmill speed by 2 m every 2 min, at a 15° slope)	Quadriceps muscle. 0 and 3 h post‐exercise.	↑ Serum corticosterone levels in exercised mice, regardless of time↓ Fasting blood glucose in ZT12 and ZT16 upon exercise	↓ *Nr1d1* mRNA levels at 3 h after exercise sessions compared with baseline.	*Nr1d1* was positively affected by exercise, exhibiting bimodal regulation (repression following induction).
Rocha et al. (2022)	C57BL/6 mice exposed to different exercise types	Male	8 weeks of age	Acute treadmill exercise (60% of the MAP at 0% inclination for 60 min). Interval Acute treadmill exercise (alternating intensities every 5 min between 40% and 80% of the maximum aerobic capacity for 60 min). Resistance acute exercise (10 climbs with external load corresponding to 75% of body weight and 2‐min recovery between repetitions)	Gastrocnemius and soleus muscles. 0, 6, 12, and 18 h post‐exercise.	↓ Fasting blood glucose after exercise.	↓ Amplitude of the *Nr1d1* circadian rhythm in the gastrocnemius and soleus muscles of endurance and strength exercise groups compared with sedentary mice. ↓ Rev‐erb‐α nuclear protein content in the gastrocnemius muscle immediately after all exercise modalities compared with sedentary mice.	Rev‐erb‐α is extremely sensitive to physical exercise stimuli, including differentmodels and intensities in SM.
Rovina et al. (2021)	C57BL/6 mice exposed to different exercise types	Male	8 weeks of age	Acute treadmill exercise (60% of the MAP at 0% inclination for 60 min). Resistance acute exercise (10 climbs with external load corresponding to 75% of body weight and 2‐min recovery between repetitions)	Gastrocnemius and soleus muscles. 0 h post‐exercise.	No effect on fasting blood glucose after exercise.	↑ *Nr1d1* mRNA levels in the soleus muscle immediately after acute aerobic exercise compared with those of sedentary and resistance exercise.	A single session of endurance exercise can increase *Nr1d1* mRNA levels in oxidative muscle.
Pinto et al. (2022)	C57BL/6 and IL‐6 KO mice	Male	8 weeks of age	Acute exhaustive exercise on a treadmill (20 m min^−1^ at 10 degrees of inclination for 90 min)	Gastrocnemius muscle. 1 and 3 h post‐exercise	Not reported	↓ *Nr1d1* mRNA levels in the gastrocnemius muscle at 1 h and 3 h after exercise sessions in both genotypes, compared with sedentary group. ↓ *Nr1d1* mRNA levels in IL‐6 KO in basal condition.	Downregulation of *Nr1d1* was verified in the IL‐6 KO mice independent of exercise.
Stede et al. (2024)	Moderately trained young adults	Males and Females	22–42 years	Acute high‐intensity aerobic exercise (warm‐up of 10 min at 90% of GET and seven high‐intensity bouts (3 min) at 100% of RCP.	Slow and fast fibers of the Vastus lateralis muscle. 0 and 3 h post‐exercise	Not reported	↓ NR1D1 mRNA levels in slow and fast fibers at 3 h after exercise sessions relative to baseline.	High‐intensity aerobic exercise acts as a zeitgeber to reset NR1D1 inside different muscle fibers.
Popov et al. (2018)	Amateur endurance‐trained (runners, cyclists, and crosscountry skiers) exposed to different intensities.	Males	20–27 years	Acute cycling exercise (1 h at 70% or 50% VO_2max_)	Vastus lateralis muscle. 4 and 8 h post‐exercise	Not reported	↓ NR1D1 mRNA levels in the vastus lateralis muscle at 4 and 8 h after exercise sessions in both intensities, compared with baseline. ↓ *Nr1d1* mRNA levels at high‐intensity compared with low‐intensity.	NR1D1 changed expression in an intensity‐dependent manner.
Edman et al. (2024)	Healthy young adults	Males	20–22 years	Acute resistance exercise (4 sets at 7RM load with 2 min of rest using both leg press and leg extension)	Vastus lateralis muscle. 30 min, 3, 8, and 24 h post‐exercise	Not reported	↓ NR1D1 mRNA levels in the vastus lateralis muscle at 3 h and 8 h after the exercise session, compared with baseline and control individuals.	Acute resistance exercise tended to blunt NR1D1 excursions and variability.
Small et al. (2020)	Healthy young adults	Males	18–27 years	Acute cycling exercise (15 min at 80% VO_2_max)	Vastus lateralis muscle. 1 h post‐exercise	Not reported	↓ NR1D1 mRNA levels in the vastus lateralis muscle at 1 h after the exercise session relative to baseline.	NR1D1 is downregulated post‐exercise, reflecting its primary role in pathway repression.
Von Ruff et al. (2025)	Middle‐aged adults exposed to the disuse model and acute exercise.	Males and Females	50–65 years	Acute resistance exercise (4 sets of 10 repetitions at 70% of 1RM, with a 2‐min rest period between sets)	Vastus lateralis muscle. 2 h post‐exercise	↓ Knee extension strength and isometric force production after 7 days of leg suspension.	↓ NR1D1 mRNA levels in the vastus lateralis muscle at 2 h after the exercise session relative to the disused and control leg.	NR1D1 serves as an adaptive response to altered physiological demands resulting from inactivity.

*Note:* (↓) Downregulation, (↑) Upregulation.

Abbreviations: GET, gas exchange threshold; IL‐6, interleukin 6; KO, knockout; NR1D1, nuclear receptor subfamily 1 group D member 1); RCP, respiratory compensation point; RM, repetition maximum; SM, skeletal muscle; VO_2_max, maximal oxygen uptake, ZT, Zeitgeber Time.

## 7 Emerging Role of Chronic Exercise and Training Status on Upregulation of Rev‐erb‐α in the Skeletal Muscle

### 7.1 Animal Models

A pioneering study demonstrated that treadmill training (8 weeks, 5 days per week, 1–2 h per day at 8–16 m/min) significantly increased Rev‐erb‐α protein levels in the soleus and gastrocnemius muscles of wild‐type trained male mice compared to untrained controls, improving mitochondrial function and exercise performance (Woldt et al. 2013). Yasumoto et al. (2015) also confirmed an increase in *Nr1d1* gene expression in mouse skeletal muscle following 4 weeks of chronic wheel‐running activity. These trained animals displayed significant fluctuations in food intake, temperature and hormones without altering body weight, providing feedback to the central clock in the SCN and advancing the phase of circadian rhythm (Yasumoto et al. 2015). Furthermore, Sato et al. (2019) demonstrated that male trained animals (short‐term, 5 days at 6 m/min and accelerated by 2 m/min every 2 or 3 min up to 16 m/min) exhibited greater *Nr1d1* peaks than sedentary animals in the early active phase, which coincides with HIF1a activation, and consequently with adaptation of systemic energy expenditure. Thus, the molecular signature of Rev‐erb‐α differs between an acute exercise bout and long‐term training adaptation. It is plausible that chronic exercise increases Rev‐erb‐α expression due to training‐induced changes in metabolism, enzymatic activity, overall oxidative capacity, and energy demand in skeletal muscle.

Additionally, new evidence links the molecular clock to mitochondrial dynamics in skeletal muscle, suggesting that chronic exercise poses a significant physiological challenge to the clock–mitochondria axis (Carriel‐Nesvara et al. 2025; Castro‐Sepulveda 2025). For instance, aerobic exercise training (8 weeks, 5 days per week at ∼55% Vmax for ∼70 min) upregulated Rev‐erb‐α upstream target protein expression (Clock and Bmal1) and potentially promoted mitochondrial fusion by increasing Mitofusin‐2 (MFN2) levels in skeletal muscle (Pourabdi et al. 2025). However, the Rev‐erb‐α signature was not assessed, leaving its specific role unresolved. Interestingly, mice with skeletal muscle‐specific Bmal1 knockout exhibited increased Optic Atrophy 1 (OPA1) and decreased dynamin‐related protein 1 (DRP1) protein levels, indicating a tendency toward mitochondrial fusion (Ramírez‐Casas et al. 2025). Additionally, electron microscopy showed that increased mitochondrial number and mass were associated with higher Rev‐erb‐α levels in skeletal muscle (Woldt et al. 2013). Therefore, it is reasonable that Rev‐erb‐α may also mediate mitochondrial dynamics, an important beneficial adaptation promoted by regular exercise.

Chronic training at a fixed time of day might alter the pattern of muscle clock gene expression. In this context, aerobic exercise (12 weeks at 12 cm/s for 10 min daily at 0% incline, representing moderate intensity, performed five times a week at 09:00) increases *Nr1d1* in the tibialis anterior, though with limited effects in female animals (Zumbro et al. 2026). Women may generally have an earlier chronotype than men (Fischer et al. 2017), which could explain these results. Furthermore, the exercise is performed during the rest phase, thereby restricting NR1D1 modulation.

Disruption of circadian rhythms substantially increases the risk of cancer development (Clemente‐Suarez et al. 2025). However, regular exercise can positively affect clock gene regulation and, potentially, counteract the progressive effects of the tumor. In this scenario, de Souza Teixeira et al. (2023) reported that male mice inoculated subcutaneously with Lewis lung carcinoma cells and exposed to aerobic training (forced treadmill running, 3 weeks, sessions of 40–60 min of training at 60% of maximum speed) at random times of the day had higher *Nr1d1* mRNA levels compared to the sedentary cancer group (de Souza Teixeira et al. 2023). These effects were accompanied by a reduction in tumor weight and changes in myokine expression toward an anti‐inflammatory profile in the gastrocnemius muscle, which were not observed in the cancer‐sedentary group. Therefore, exercise may serve as a potent Zeitgeber in cancer, influencing the circadian clock system in peripheral tissues through immunometabolic circuits.

Since the aging process causes significant impairment of muscle structure and function, Yao et al. 2024 explored the potential benefits of endurance training (8 weeks, 5 days per week at 12–15 m/min) to alleviate sarcopenia phenotypes by atrophy‐related factors and *Nr1d1* in the skeletal muscle of prematurely aging mice (SAMP8 model). The authors found that the JAK2/STAT3 pathway was downregulated in the *vastus lateralis* of male trained SAMP8 compared with sedentary SAMP8. At the same time, *Nr1d1* mRNA levels and Rev‐erb‐α protein content were significantly increased, contributing to increasing muscle strength, endurance, and muscle CSA. More recently, Vitorino et al. (2025) observed increased muscle performance and *Nr1d1* mRNA expression, while *Arntl* decreased in the gastrocnemius of middle‐aged male mice following both low (below the anaerobic threshold) and high‐intensity chronic swimming exercise, emphasizing the potential role of chronic exercise in influencing clock genes during aging (Vitorino et al. 2025). Overall, these studies suggest that targeting NR1D1 in aging is promising for achieving therapeutic benefits by improving or correcting circadian rhythms.

Contrary to these previous data, Lee et al. (2022) did not observe changes in Nr1d1 mRNA levels in the skeletal muscle of old male animals in response to aerobic exercise (4 weeks of treadmill exercise, 3 days per week with 30 min per session, at 12–20 m/min). In addition to this observation, the authors reported lower gene expression in the soleus and gastrocnemius muscles of young sedentary mice than in young exercised mice. However, the authors have not addressed the interval between the last exercise session and tissue collection, thereby obscuring the potential time interference in Rev‐erb‐α levels (Lee et al. 2022). Furthermore, they used only RNA‐seq and a single time point, without validating the findings by RT‐qPCR or protein levels. Furthermore, *Nr1d1* can exhibit gene expression variations that depend on the timing of measurement, tissue, and species; hence, these factors must also be considered.

Our laboratory recently reported that acute exhaustive exercise and chronic excessive training altered Rev‐erb‐α levels in mouse skeletal muscle, in parallel with increased muscle fatigue and impaired physical performance (da Rocha et al. 2025). Both protocols increased kynurenine 3‐monooxygenase (KMO) expression and reduced kynurenine aminotransferase 1 (KAT1) expression. KMO directs kynurenine toward the 3‐hydroxykynurenine branch, whereas KAT1 contributes to kynurenic acid formation. In male mice subjected to exhaustive exercise, Rev‐erb‐α and KMO protein levels were inversely correlated. These alterations were accompanied by a loss of detectable Rev‐erb‐αcircadian rhythmicity and by an enzyme‐expression pattern consistent with preferential routing of kynurenine toward the KMO‐associated branch. However, because downstream kynurenine metabolites and neurotoxicity‐related outcomes were not directly assessed, these findings do not establish a neurotoxic profile. The inverse association between Rev‐erb‐α and KMO raises the hypothesis that Rev‐erb‐α may regulate components of the kynurenine pathway; nevertheless, direct transcriptional repression and causal involvement in fatigue or impaired performance remain to be demonstrated. Thus, Rev‐erb‐αrepresents a candidate mechanistic target for investigating maladaptive responses to exhaustive exercise and excessive training rather than an established therapeutic target.

### 7.2 Human Studies

The ability of exercise to chronically induce Rev‐erb‐α expression in human muscle has not been conclusively demonstrated, although a scientifically plausible rationale exists. Human primary myotubes of obese and Type 2 diabetes patients displayed dampened rhythmicity compared to those of lean individuals. In contrast, male young endurance‐trained athletes had higher Rev‐erb‐α amplitude than sedentary subjects and individuals with metabolic diseases (Hansen et al. 2016). Furthermore, rhythmicity in nicotinamide phosphoribosyltransferase (NAMPT) and sirtuin 1 (SIRT1) gene expression was only observed in trained athletes. Similarly, Rev‐erb‐α expression in human mononuclear cells was significantly associated with physical activity and natural daylight exposure, corroborating animal data and skeletal muscle responsiveness (Gubin et al. 2025).

In a longitudinal single‐leg design, overweight and obese men with insulin resistance completed 12 weeks of high‐intensity interval training, with sessions consistently performed in the afternoon. Although training increased mitochondrial respiration in the vastus lateralis, NR1D1 mRNA levels remained unchanged (Harmsen et al. 2024). Because the study did not include a non‐exercise control group, the observed changes cannot be attributed exclusively to the training intervention. Taken together with previous studies, these findings indicate that the effects of chronic exercise on skeletal muscle NR1D1/Rev‐erb‐α in humans are heterogeneous rather than uniformly inductive. Direct comparisons among aerobic, resistance, and combined training protocols are needed to determine how exercise modality, training timing, and participant characteristics influence Rev‐erb‐α ‐related responses in human skeletal muscle. Table 2 summarizes the effects of chronic exercise models on skeletal muscle NR1D1/Rev‐erb‐α in animals and humans.

**Table 2:** Effects of chronic exercise on Rev‐erb‐α signature modulation in rodents and humans.

Reference	Experimental model and condition	Sex	Age	Exercise dose (frequency, duration, intensity)	Tissue and time analyses	Skeletal muscle phenotype and metabolic effects	Rev‐erb‐α detection assay and findings	Conclusions on Rev‐erb‐α function
Woldt et al. (2013)	C57BL/6 mice	Male	Not reported	Treadmill training (8 weeks, 5 days per week, 1–2 h per day at 8–16 m/min)	Soleus and gastrocnemius muscles. Time not reported	↑ Mitochondrial biogenesis and function in SM, as well as exercise capacity in trained mice	↑ Rev‐erb‐α protein content in trained mice compared with sedentary mice.	Rev‐erb‐α improves muscle oxidative function by regulating mitochondrial number and function.
Yasumoto et al. (2015)	BALB/c mice	Male	5 weeks of age	Chronic wheel‐running activity (4 weeks)	Muscle type not reported ZT0–24	↑ Plasma corticosterone levels at ZT10 and ↓ leptin levels during ZT18 to ZT6 in trained mice	↑ Peak of *Nr1d1* over 24 h in the SM of trained mice.	*Nr1d1* provides feedback to the central circadian clock, which systemically governs behavioral and physiological rhythms during exercise.
Sato et al. (2019)	C57BL/6 mice	Male	10–11 weeks of age	Short‐term of treadmill training (5 days at 6 m/min and accelerated by 2 m/min every 2 or 3 min up to 16 m/min)	Gastrocnemius and quadriceps muscles ZT0–24	↑ Metabolic impact of morning exercise (start of active phase) than at night (start of rest phase) in trained mice, leading to more carbohydrate and ketone use, and lipid degradation.	↑ Peak of *Nr1d1* over 24 h in the SM of trained mice.	Higher *Nr1d1* peaks align with HIF1a activation and systemic energy adaptation.
Zumbro et al. (2026)	Fisher 344 × Brown Norway rats	Male and female	24 months of age	Treadmill training (12 weeks, 3 days per week, sessions at 12 cm/s for 10 min daily at 0% incline	Tibialis anterior and soleus muscles 20.00 h (ZT2)	↑ Time to exhaustion and relative grip strength only in male‐trained rats	↑ *Nr1d1* mRNA levels in the tibialis anterior of male trained rats	*Nr1d1* gene expression can depend on sex and the skeletal muscle analyzed.
de Souza Teixeira et al. 2023	C57BL/6 mice inoculated with Lewis lung carcinoma cells	Male	8–10 weeks of age	Treadmill training (3 weeks, 5 days per week, sessions of 40–60 min at 60% of maximum speed)	Gastrocnemius muscle ZT0–24	↑ Muscle weight, corticosterone AUC concentration, and myokines production in the trained groups	↑ Peak of *Nr1d1* in the gastrocnemius muscle of trained mice compared to the sedentary cancer group	Exercise is a potent Zeitgeber and may thus contribute to changes in *Nr1d1* expression that reduce tumor weight.
Yao et al. (2024)	SAMR1 and SAMP8 mice	Male	7 months of age	Treadmill training (8 weeks, 5 days per week at 12–15 m/min)	Gastrocnemius muscle and vastus lateralis 24 h after last exercise session	↑ Muscle coordination, grip strength, and gastrocnemius CSA in SAMP8‐trained mice	↑ Rev‐erb‐α protein content and *Nr1d1* mRNA levels in the gastrocnemius of SAMP8 mice compared to sedentary SAMP8 mice.	Endurance training improved sarcopenia phenotypes, with Rev‐erb‐α upregulation as a potential mechanism.
Vitorino et al. (2025)	C57BL/6 mice exposed to different exercise intensities	Male	12 months of age	Swimming training (12 weeks, 3 days per week) with low group had 40‐min sessions with loads corresponding to 4% of the BW, while the high group had 20‐min sessions with 6% of the BW	Gastrocnemius muscle 72 h after last exercise session	↑ Muscle load and GLUT4 content in the gastrocnemius muscle. ↓ Fasting blood glucose are independent of exercise intensity	↑ *Nr1d1* mRNA levels in the gastrocnemius muscle of both trained groups compared to the sedentary group	Chronic exercise modulating *Nr1d1* opens the possibility of improvements in exercise capacity and metabolism following low‐intensity regimens.
Lee et al. (2022)	Young and old C57BL/6 mice	Male	9 or 84 weeks of age	Treadmill training (4 weeks, 3 days per week with 30 min per session, at 12–20 m/min).	Gastrocnemius and soleus muscle. Time not reported.	Not reported	↓ *Nr1d1* mRNA levels in the gastrocnemius and soleus muscles of the young trained group compared to the young sedentary group. No effects of exercise on the *Nr1d1* mRNA levels of old mice.	Nr1d1 shows distinct results in the exercise and aging context.
Rocha et al. 2025	C57BL/6 mice were exposed to overtraining.	Male	7 weeks of age	Overtraining (8 weeks, 5 days per week in downhill running at 75% of the MAP	Gastrocnemius muscle ZT0–12	↑ Muscle fatigue in the overtraining group. ↓ Body weight and maximum aerobic power in the overtraining group.	↓ pRev‐erb‐α/total Rev‐erb‐α protein content in overtraining group at ZT12 compared to sedentary group	Overtraining abolishes Rev‐erb‐α circadian rhythm and shifts to a neurotoxic profile in mice
Hansen et al. (2016)	Human primary myotubesfrom Young endurance‐trained athletes, young lean sedentary, obese subjects, and Type 2 diabetic patients.	Male	±22 years (young and sedentary athletes) ±57 years (obese) ±59 years (Type 2 diabetic patients)	Endurance‐trained athletes were performing exercise (long‐distance running, cycling, swimming) at least three times a week for the past 2 years and had a VO_2_max > 55 mL/min/kg	Vastus lateralis muscle 0–72 h.	↑Insulin sensitivity in young and athletes compared to obese and Type 2 diabetic patients	↑ NR1D1 amplitude over 72 h in young and athletes compared to obese and Type 2 diabetic patients	Human skeletal muscle NR1D1 circadian rhythms are affected in donors with a sedentary lifestyle andpoor health status
Harmsen et al. (2024)	Overweight and obese humans	Male	40–75 years	High‐intensity interval training (12 weeks, 3 times per week with sessions performed by 10 intervals of 1 min at 80%–90% of an individual's Wmax on a cycle ergometer)	Vastus lateralis muscle 8.00, 13.00, 18.00, and 23.00 h.	↓ 24‐h plasma glucose in the trained group. ↑ Mitochondrial respiration in SM of the trained group was independent of time of day	No effects of exercise on the NR1D1 mRNA levels of the trained group. ↑ NR1D1 mRNA levels at 23:00 compared with 18:00 and 08:00, independent of chronic exercise exposure	Limited effects of High‐intensity interval training on NR1D1 response

*Note:* (↓) Downregulation, (↑) Upregulation.

Abbreviations: AUC, area under the curve; BW, body weight; CSA, cross‐sectional area; HIF‐1α, hypoxia‐inducible factor‐1 alpha; KO, knockout; MAP, maximum aerobic power; NR1D1, nuclear receptor subfamily 1 group D member 1; RM, repetition maximum; SAMP8, senescence accelerated mouse‐prone 8; SM, skeletal muscle; VO_2_max, maximal oxygen uptake; Wmax, maximum workload.

Nevertheless, studies suggest a clinically significant association between the NR1D1 transcript signature and musculoskeletal dysfunction in humans. Notably, one study found that a random forest model identified NR1D1 as a critical biomarker for predicting sarcopenia (Zhang et al. 2025). Furthermore, NR1D1 mRNA and protein levels were significantly reduced in human osteoarthritis compared with healthy individuals, thereby impairing transforming growth factor‐beta (TGF‐β) signaling, a key pathway in cartilage remodeling and homeostasis (Akagi et al. 2017). Importantly, atherosclerotic peripheral arterial disease leads to limb ischemia, muscle damage, and reduced lower‐limb mobility. Zhu 朱培 et al. (2025) found disrupted circadian clock gene expression, resulting in decreased NR1D1 mRNA levels in the gastrocnemius muscle of patients with peripheral artery disease, suggesting that Rev‐erb‐α is important for muscle function and recovery. Thus, targeting NR1D1 through structured exercise interventions holds clinical and therapeutic promise (Zhu 朱培 et al. 2025).

## 8 Inherent Relationship Between Rev‐erb‐α and Physical Performance

Mice lacking *Nr1d1* exhibited cognitive impairment, particularly in tasks involving contextual memory and the ability to adapt to repeated stimuli. They also performed worse on the nest construction test (Goto et al. 2017). Additionally, this genetic deficiency disrupts key processes in brain development, such as neuronal migration, axon formation, and dendrite growth, which likely contribute to their reduced physical capabilities and muscle performance (Jager et al. 2014). More recently, our laboratory showed that chronic aerobic exercise (8 weeks, 4 sessions/week) upregulated *Nr1d1* and Rev‐erb‐α protein levels in the hippocampus of an Alzheimer's disease animal model. Furthermore, we observed a positive correlation between Nr1d1 and motor coordination and memory improvement, which reinforces the hypothesis of its involvement in functional capacity and health (Morais et al. 2025).

In animal model studies, mice lacking Rev‐erb‐α exhibited marked impairments in spontaneous locomotion and muscle force generation, with reductions of approximately 50% and 90%, respectively (Boulinguiez et al. 2022; Woldt et al. 2013). These findings indicate that *Nr1d1* is essential for maintaining proper musculoskeletal function. While resting oxygen consumption (VO_2_) was similar between rodents, Rev‐erb‐α‐deficient mice demonstrated a markedly lower VO_2_max during exertion, leading to a more than 60% drop in aerobic exercise capacity. When animals are exposed to an endurance test at 70% of their individual VO_2_max, half of the Rev‐erb‐α knockout mice ceased running within 50 min compared to just 20% of wild‐type mice, highlighting their limited exercise endurance (Woldt et al. 2013). Overall, the knockout group ran shorter distances and for less time than their littermate controls. Also, pharmacological activation of Rev‐erb‐α in mice with the synthetic ligand SR9009 improved exercise capacity. Notably, in an endurance exercise test, mice treated with the Rev‐erb‐α agonists displayed significantly longer time and distance than vehicle‐treated mice (Woldt et al. 2013), suggesting that these agonists can act as exercise mimetics or enhancers.

According to the 2026 World Anti‐Doping Agency Prohibited List, REV‐ERB agonists, including SR9009 and SR9011, are prohibited at all times under S4: Hormone and Metabolic Modulators (Geldof et al. 2016). These experimental compounds have sometimes been marketed as exercise mimetics because preclinical studies have reported effects on energy metabolism and exercise capacity. However, evidence supporting their performance‐enhancing effects is derived predominantly from animal studies, and their efficacy and long‐term safety in humans have not been established.

Although a direct link between NR1D1 and muscle performance in human skeletal muscle has not been established, researchers have observed elevated NR1D1 mRNA expression in effector memory CD4^+^ T cells of master athletes following an acute bout of exercise. This rise in expression was positively associated with greater VO_2_max, longer exercise duration, and higher power output (de Souza Teixeira et al. 2021). Interestingly, this pattern was absent in healthy individuals who had not engaged in regular or competitive physical activity for two decades. Supporting this, male rugby players had significantly higher NR1D1 gene expression in their blood than their sedentary peers (Song et al. 2018), reinforcing the impact of regular exercise on NR1D1 levels. Figure 5 summarizes acute, chronic, physical activity levels, phase and adverse response in the Rev‐erb‐α signature.

**Figure 5:**
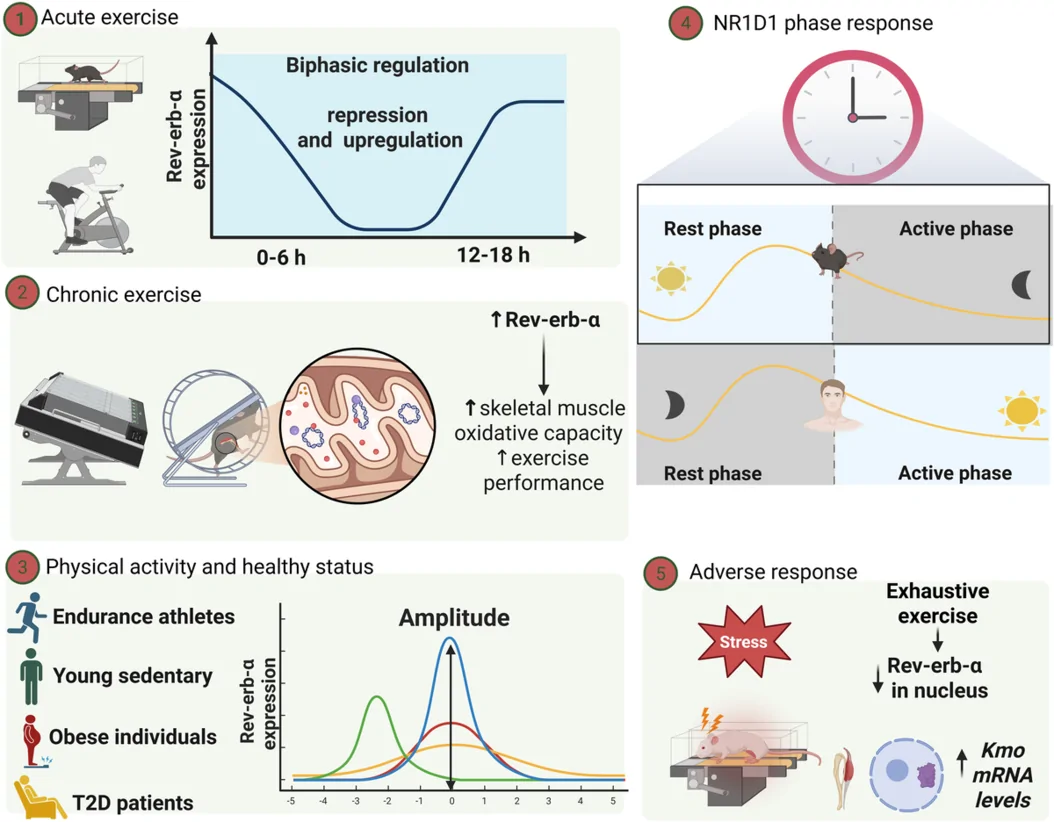
Summary of Rev‐erb‐α signature in acute, chronic exercise, and physical activity status. (1) In the acute response, Rev‐erb‐α repression was observed immediately post‐exercise, accompanied by a subsequent increase until 18 h, which reflects its primary role in pathway repression. (2) In chronic exercise, upregulation of Rev‐erb‐α can act as an adaptive mechanism that induces robust responses in energy demand and mitochondrial function to coordinate oxidative capacity and muscle performance. (3) Young endurance‐trained athletes had higher Rev‐erb‐α amplitude when compared to sedentary controls and individuals with metabolic diseases. (4) Rev‐erb‐α expression peaks during the rest phase and declines to its lowest point during the active phase in rodents and humans. (5) Animals exposed to exhaustive exercise exhibited a negative correlation between Rev‐erb‐α and Kmo protein levels, abolishing circadian rhythm and shifting the neurotoxic profile. h, hours; Kmo, kynurenine 3‐monooxygenase; mRNA, messenger ribonucleic acid; NR1D1, nuclear receptor subfamily 1 group D member 1, encoding REV‐ERBα; REV‐ERBα, nuclear receptor REV‐ERB alpha; T2D, Type 2 diabetes. Biorender® web‐based software was used to create the figure.

## 9 Exercise Timing Determines the *Nr1d1* Signature and Downstream Phenotypes in Skeletal Muscle

According to studies summarized in this review and publicly available data on CircaDB (Pizarro et al. 2013), *Nr1d1* mRNA levels typically peak during the middle to late rest (light) phase in rodents. In a standard 12:12 light‐dark cycle where “Lights On” is at ZT0 (Zeitgeber Time 0), the peak usually occurs between ZT4 and ZT8. In contrast, the lowest expression occurs during the middle‐to‐late active (dark) phase, typically between ZT16 and ZT22. However, protein levels show a slight delay (about 2–4 h) because it takes time for the cell to translate the mRNA into a functional protein.

In rodent studies, exercise performed during the active phase has been associated with greater engagement of glycolytic and stress‐responsive pathways and less pronounced induction of oxidative and mitochondrial gene programs (S. Sato et al. 2019). This response pattern may be consistent with increased REV‐ERBα–HDAC3 corepressor activity at metabolic loci, based on mechanistic evidence from related experimental models (Feng et al. 2011; Gerhart‐Hines et al. 2013). Conversely, exercise during the rest phase, when skeletal muscle *Nr1d1* expression is relatively high, has been associated with greater activation of PGC‐1α‐related transcriptional programs and oxidative metabolic responses (Dyar et al. 2014; S. Sato et al. 2019). Collectively, these findings suggest that exercise timing may modulate Nr1d1/Rev‐erb‐α‐associated transcriptional signatures and downstream metabolic responses in skeletal muscle. However, this proposed model integrates evidence derived predominantly from rodent studies and should be considered hypothesis‐generating, because direct causal regulation by Rev‐erb‐α in human skeletal muscle has not been demonstrated.

The optimal timing of exercise for maximizing skeletal muscle performance and metabolic adaptation remains debated. Exercise acts as a non‐photic zeitgeber that can shift peripheral circadian clocks and modulate metabolic and circadian outputs (Hesketh et al. 2024). Rodent studies indicate that timed exercise can phase‐shift the skeletal muscle clock without detectably altering the SCN under the experimental conditions examined, supporting the possibility of tissue‐specific entrainment (Procopio and Esser 2025; Zhe Zhang et al. 2025). Because civil‐time terms such as “morning” and “evening” do not correspond uniformly to biological phases across species, exercise timing in rodents is more appropriately described relative to the active/wake and rest/sleep phases. Liu et al. (2025) reported that low‐intensity exercise performed near the end of the active phase, before the transition to the rest phase, produced greater reductions in body mass and improvements in muscle strength, endurance, and glucose tolerance than exercise performed near the beginning of the active phase. Consistently, other studies in mice reported greater improvements in muscle endurance and body mass when exercise was performed during the early rest or late active phases than during the late rest or early active phases (Dalbram et al. 2019; Hesketh et al. 2023).

Long‐term exercise does not produce timing‐dependent effects on body composition in Rev‐erbα/β (Rev‐MKO) muscle‐specific knockout mice, despite normal feeding habits (Liu et al. 2025). This suggests a Rev‐erb‐dependent anticipatory mechanism rather than a diet‐responsive one, which is essential for the daily fluctuations in systemic metabolic adaptations to exercise. Specifically, the multiomics approach indicates that muscular bioenergetics is intrinsically modulated by Rev‐erbα/β. As *Nr1d1* levels rise in the sleep phase, signaling the upcoming wake cycle, depleting Rev‐erb enhances lipid oxidation and conserves glucose, especially before sleep (Liu et al. 2025). The absence of two Reverb proteins in skeletal muscle increases fatigue resistance and reduces strength, as observed before sleep (ZT23‐1). This deletion promotes a preference for fatty acid utilization and likely suppresses the training‐induced upregulation of carbohydrate metabolism and the subsequent anabolic remodeling of muscle contractile component machinery. Thus, exercise timing may determine the NR1D1 transcriptional signature by aligning molecular signaling with circadian phases of NR1D1‐mediated repression, thereby biasing skeletal muscle adaptation toward glycolytic or oxidative phenotypes.

Exercise performed between late morning and early evening coincides with improved human muscle performance (Hesketh and Esser 2024) and, consequently, reinforces REV‐ERBα–dependent regulatory programs in skeletal muscle. Consistent with this model, studies show that the active phase elicits transcriptional and metabolic responses that favor glycolytic capacity and acute muscle performance, whereas exercise performed closer to phases of NR1D1 modulation may enhance oxidative metabolic pathways and mitochondrial remodeling in skeletal muscle (Ezagouri et al. 2019).

NR1D1 expression can remain suppressed for up to 6 h after acute exercise. During this suppression, it is important to avoid exercise stimuli or increased intensity and allow expression to return to normal. Thus, aligning exercise time with the endogenous NR1D1 peak so that high levels associated with chronic exercise can compensate for the acute reduction. At the same time, species‐specific activity patterns define the optimal temporal window for reinforcing NR1D1‐dependent metabolic regulation. However, studies conducted within 24 h of the acute exercise session make it difficult to distinguish enduring changes in clock output from the immediate transcriptional response to exercise.

In humans, which are typically diurnal, the circadian phase of NR1D1 expression is shifted relative to that in rodents. However, Harmsen et al. 2024 observed a peak in NR1D1 at 23:00 compared with 18:00 and 08:00 independent of chronic exercise exposure, corroborating findings from the rest phase in animals. Interestingly, this response coincided with a physiological shift toward increased energy expenditure and carbohydrate oxidation. Thus, NR1D1 regulates the daily switch between fuel sources, and its peak favors energy homeostasis in humans.

Muscle performance varies between “larks” and “owls” athletes (morningness/eveningness circadian types), with phenotype and time since awakening serving as indicators of how well the circadian system is entrained and aligned with internal biological time (Z. Zhang et al. 2026). These are all factors that need to be carefully considered in future studies. It is important to emphasize that for the general population, exercising at nearly any time of day is better than being sedentary. Integrating the individual NR1D1 signature into exercise prescriptions is crucial for maximizing health benefits and optimizing performance. This approach represents a shift from “one‐size‐fits‐all” routines to “precision exercise” that aligns with the body's natural 24‐h cycle. NR1D1 offers a promising integrative approach, providing innovative strategies to promote health, prevent disease, and optimize athletic performance. In practice, athletes should align their regular training times with peak NR1D1 periods and the performance demands of competitions. However, the peak performance and optimal training effects may not always coincide.

## 10 Methodological Limitations, Future Viewpoints, and Challenges

A reverse light‐dark cycle can be used with mice in research settings to align the animal's active period with the human workday, facilitating easier and more efficient experimental work. This approach allows researchers to work with mice during their natural active phase (typically night‐time) while minimizing disruptions to their circadian rhythm. However, some studies evaluating Reverb‐α do not report how the animal cycle was performed, limiting our biological understanding. Chronic exercise at a fixed time of day alters the oscillatory pattern of muscle clock gene expression, adding another layer of complexity that warrants further investigation.

To reliably detect genuine NR1D1 patterns at transcriptional and translational levels, conducting studies with a larger number of biological replicates and extended time points, combined with robust statistical approaches, is essential. A key limitation of the experiments discussed in this review is that only a small number adhered to the established guidelines for analyzing rhythmic data and ensuring sufficient statistical power, which recommend a minimum of 12 time points per cycle throughout two complete cycles (Hughes et al. 2017). This robust repeated measurement design strategy can help separate short‐term responses to exercise from longer‐term adaptations. Additionally, fasting affects core clock genes and proteins, resulting in blunted rhythmicity of Rev‐erb‐α in the skeletal muscle, which makes it a relevant issue for outcomes variability (Kinouchi et al. 2018).

In circadian research, the *p*‐value of the frequentist statistic alone may not be sufficient to capture the full picture of rhythmic fluctuations over 24 h (Y. Zhang et al. 2025). Analytical tools include parametric techniques, such as cosinor and Lomb‐Scargle models, as well as non‐parametric methods such as ARSER, JTK CYCLE, and RAIN, which should be used critically in each situation. Also, Bayesian hierarchical methods and MetaCycle are integrative tools that aggregate results from these methods (G. Wu et al. 2016; Y. Zhang et al. 2025).

Most animal studies investigating the isolated loss of Rev‐erb‐α have used global Nr1d1 knockout mice, i.e., models in which Nr1d1 is deleted throughout the body. Evidence from skeletal muscle‐specific models remains limited and currently includes primarily the muscle‐specific double knockout of Nr1d1 and Nr1d2 (REV‐ERBα/β) described by Liu et al. (2025). Consequently, the effects of deleting Nr1d1 alone specifically in skeletal muscle remain insufficiently characterized. Muscle‐specific deletion may help distinguish tissue‐autonomous effects from systemic influences and compensatory responses originating in non‐muscle tissues. Systematic comparisons of Nr1d1/Rev‐erb‐α abundance across muscles with different fiber‐type compositions, such as the soleus, extensor digitorum longus, gastrocnemius, and quadriceps, following chronic exercise also remain limited. Given the reported higher abundance of Rev‐erb‐α in Type I fibers and the fiber‐type remodeling associated with several muscle disorders, future studies should determine whether Nr1d1/Rev‐erb‐α responses to different exercise modalities vary between Types I and II fibers and among skeletal muscles.

In vitro approaches focused on silencing Rev‐erb‐α in C2C12. Still, the CRISPR‐Cas9 technique is necessary to improve efficiency, specificity, and heritability, given the transient, cytoplasmic nature of knockdown or RNAi technologies (Barrangou et al. 2015). Additionally, the emergence of three‐dimensional skeletal muscle models, such as spheroids and organoids, can offer new experimental opportunities to study Rev‐erb‐α dynamics under physiologically relevant conditions. These 3D systems can integrate muscle fibers, satellite cells, and even neural or immune components within a structured microenvironment, more closely mimicking human muscle physiology than conventional 2D culture (Mavrommatis et al. 2023). Functional human skeletal muscle organoids have been successfully generated from pluripotent stem cells, enabling the investigation of differentiation, metabolism, and contractile function (Park et al. 2025; Yang et al. 2025).

More recently, Liu et al. (2025) reported that mice with skeletal muscle‐specific double knockout of Nr1d1 and Nr1d2 (Rev‐erb‐α/β; REV‐MKO mice) exhibited increased endurance capacity at low running speeds but not at high running speeds. This muscle‐autonomous phenotype appeared to involve bioenergetic adjustments rather than changes in muscle morphology, because the combined deletion did not alter baseline mitochondrial content or myofiber CSA. Metabolic measurements indicated that REV‐ERBα/β signaling collectively suppresses fatty acid oxidation and favors carbohydrate metabolism near the transition from the active phase to the rest phase (Liu et al. 2025).

These findings suggest that skeletal muscle Rev‐erb‐α/β contributes to the time‐dependent coordination of energy demand, substrate utilization, and aerobic performance. However, because both receptors were deleted, this model does not distinguish the specific contribution of Nr1d1/Rev‐erb‐α from that of Nr1d2/Rev‐erb‐β.

Although the double KO experiment is powerful for studying gene function (Liu et al. 2025), it may have notable disadvantages. These include the potential for unforeseen consequences resulting from the simultaneous removal of multiple genes and the difficulty in interpreting results when genes are functionally distinct. In this line, Rev‐erb‐β‐deficient mice show differential expression of mitochondrial and lipid metabolism genes in muscle compared to Rev‐erb‐α‐deficient mice (Amador et al. 2018). Amador et al. (2018) observed a more oxidative profile in Rev‐erb‐β global KO mice. Still, Rev‐erb‐α global KO mice showed a shift towards a less oxidative phenotype, as evidenced by downregulation of *Ppargc1a*, *Cpt1b*, *Acadl*, *Acads*, *Ucp2*, *Ucp3*, *Myh2*, and *Tpm3*. Thus, deletion of different Rev‐erb isoforms can have opposite effects, masking the individual gene's contribution to the animal phenotype.

Sex influences circadian rhythm regulation, suggesting that biological differences between males and females affect sleep cycles, internal clock timing, and physiological functions. Testosterone appears to reduce *Nr1d1* gene expression, whereas estradiol tends to increase it (Noh et al. 2022). Notably, most investigations of Rev‐erb‐α in skeletal muscle used only male animals or subjects, yet its expression varies by sex during liver aging, with higher levels in older male rats than in females (Noh et al. 2022). On the other hand, older human females exhibit significantly greater NR1D1 mRNA levels in skin cells than older males, and young females have lower levels than their older counterparts (Cvammen et al. 2024). Despite these findings, there remains a lack of information on how the Rev‐erb‐α signature changes in response to physical exercise across skeletal muscles in males and females of different ages. Understanding these sex‐based differences across the life course is essential for designing tailored exercise interventions and optimizing individual outcomes.

It is essential to note that previous investigations have only examined Rev‐erb‐α phosphorylation in response to exercise. Furthermore, chronic exercise has not established the specific biological function of Rev‐erb‐α in different cellular compartments (cytosol and nucleus). Without investigating other posttranslational modifications, our understanding of how Rev‐erb‐α functions within the musculoskeletal system remains incomplete. Additionally, a full omics cascade, including genomics, transcriptomics, and proteomics in a holistic view, as well as cutting‐edge techniques such as single‐cell omics and spatial sequencing, is necessary to study adaptations in the specific skeletal muscle of Nr1d1 knockout mice following exercise. Expanding the molecular landscape of muscle through these multi‐omics strategies could reveal molecular hubs, key complex mechanisms and novel biomarkers and pathways, ultimately guiding precision interventions to boost muscle health and athletic performance.

In human studies, research involving Rev‐erb‐α is limited to small participant groups, and the main findings still need to be confirmed in more diverse clinical populations. The relationship between the level of Rev‐erb‐α expression in response to different doses and the resulting clinical effects remains unclear. Moving forward, it is important to explore how individuals differ in their responsiveness to Rev‐erb‐α, with some showing robust adaptations (high responders) and others displaying minimal changes (low responders). This information could help uncover key influences, such as genetic background, initial gene expression levels, and the distinct molecular pathways activated by exercise. Even small variations in Rev‐erb‐α expression could yield meaningful training benefits, underscoring its potential role in personalized muscle health strategies.

The NR1D1 drug target has beneficial effects in preclinical studies, including reducing chronic inflammation and apoptosis while regulating metabolic pathways. Despite this potential, clinical development has progressed slowly, primarily due to challenges such as limited drug safety profiles, poor absorption and distribution efficiency, and species‐specific differences in circadian regulation. It is essential to distinguish the specific and exclusive effects of NR1D1 agonists from the physiological benefits of chronic exercise.

## 11 Conclusions and Outlook

Accumulating evidence identifies Rev‐erb‐α as a component of the accessory feedback loop of the molecular circadian clock and as a regulator of transcriptional programs relevant to skeletal muscle. Genetic loss‐ and gain‐of‐function studies in cultured muscle cells and animal models provide causal evidence that manipulation of Nr1d1/Rev‐erb‐α can alter myogenesis, muscle mass regulation, tissue remodeling, calcium homeostasis, and autophagy, although findings from global knockout models do not establish muscle‐autonomous effects. Observational studies further associate NR1D1/Rev‐erb‐α expression and rhythmicity with metabolic and functional phenotypes in skeletal muscle. Pharmacological studies have also reported changes in these processes following treatment with REV‐ERB modulators; however, these findings require cautious interpretation because currently available compounds may lack isoform specificity and exert Rev‐erb‐α ‐independent effects. Overall, Rev‐erb‐α is a multifunctional nuclear receptor associated with context‐dependent and pleiotropic regulation of skeletal muscle physiology.

Acute and chronic exercise have been associated with context‐dependent changes in skeletal muscle NR1D1/Rev‐erb‐α. A biphasic temporal pattern, characterized by an immediate post‐exercise reduction followed by recovery toward baseline or a later increase, has been reported in some acute‐exercise studies; however, this response should not be considered universal. Chronic training has also been associated with changes in NR1D1/Rev‐erb‐α expression and rhythmicity that may contribute to the coordination of energy metabolism, mitochondrial function, and oxidative capacity, although the direction and magnitude of these responses vary across exercise protocols and study populations. Observational findings further indicate greater Rev‐erb‐α circadian amplitude in physically active or trained individuals than in sedentary individuals or some populations with metabolic disease. Collectively, these findings identify NR1D1/Rev‐erb‐α as a potential mediator and biomarker of exercise‐related metabolic adaptation, but they do not establish that chronic Rev‐erb‐α upregulation directly causes metabolic improvements or represents a validated therapeutic target.

Despite the emerging role of higher Rev‐erb‐α levels in improving physical performance in animals, the impact of chronic exercise on human muscle and specific biological functions across different cellular compartments remains rudimentary. Furthermore, the particular implications of muscle‐specific Rev‐erbα knockout remain unclear. Additionally, the standardization of the time course, fasting conditions, and statistical methods must be rigorously controlled (Figure 6). Notably, Rev‐erb‐α overexpression has been associated with reduced activation of muscle atrophy pathways and fibrosis, preserving muscle mass and contractile function. However, given Rev‐erb‐α's dual roles in muscle cell differentiation and phenotype regulation, it is crucial to determine optimal Rev‐erb‐α levels across different contexts to guide the development of therapies that promote muscle growth or prevent muscle wasting. Understanding the complex mechanisms underlying the regulation and action of the Rev‐erb‐α protein may offer new insights for improving muscle function across diverse health conditions and diseases.

**Figure 6:**
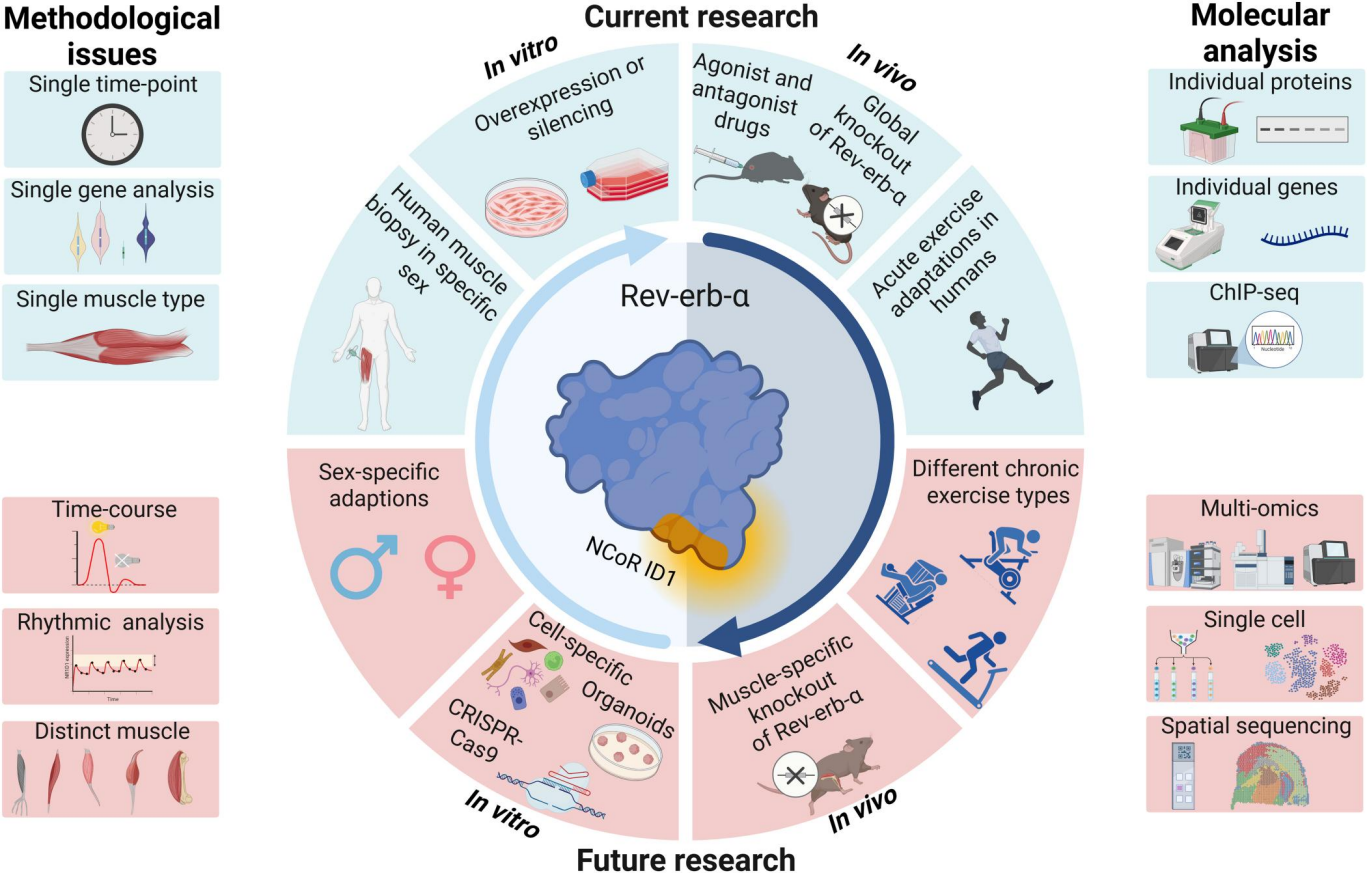
Advancing Rev‐erb‐α research: From current studies to future translational applications. The figure contrasts the current state of methods, data analysis, and molecular approaches (blue) with future research (red). In vitro approaches have investigated silencing or overexpression of Rev‐erb‐α in C2C12 cells. Still, three‐dimensional skeletal muscle models, such as spheroids and organoids, offer new experimental opportunities to study Rev‐erb‐α dynamics. Furthermore, the studies focused on pharmacological modulators, global KO and acute exercise in human skeletal muscle. As reported below, the impact of muscle‐specific Rev‐erb‐α knockout, as well as the effects of cell‐ and sex‐specific adaptations and different exercise modalities in humans, must be established. The investigations focused on a single time‐point, a single analysis, and a unique muscle. Future approaches can advance across multiple tissues and robust statistical analysis using multi‐omics, single‐cell, or spatial sequencing. ChIP‐seq, chromatin immunoprecipitation followed by sequencing; CRISPR–Cas9, clustered regularly interspaced short palindromic repeats–CRISPR‐associated protein 9; ID1, interaction domain 1; NCoR, nuclear receptor corepressor; NR1D1, nuclear receptor subfamily 1 group D member 1; REV‐ERBα, nuclear receptor REV‐ERB alpha. Biorender® web‐based software was used to create the figure.

## Author Contributions

**Ivo Vieira de Sousa Neto:** writing – review and editing, writing – original draft, conceptualization. **Alisson Luiz da Rocha:** writing – review and editing, writing – original draft. **Ana Paula Pinto:** writing – review and editing, writing – original draft. **Eliézer Lucas Pires Ramos:** writing – review and editing, writing – original draft. **Eduardo Rochete Ropelle:** writing – review and editing, writing – original draft. **José Rodrigo Pauli:** writing – review and editing, writing – original draft. **Ellen Cristini de Freitas:** writing – review andediting, writing – original draft. **Adelino Sanchez Ramos da Silva:** writing – review and editing, writing – original draft, funding acquisition.

## Ethics Statement

This review did not involve new research with human participants or animals.

## Conflicts of Interest

The authors declare no conflicts of interest.

## Data Availability

Data sharing not applicable to this article as no datasets were generated or analysed during the current study.
